# A Context-Aware-Based Audio Guidance System for Blind People Using a Multimodal Profile Model

**DOI:** 10.3390/s141018670

**Published:** 2014-10-09

**Authors:** Qing Lin, Youngjoon Han

**Affiliations:** Electronic Engineering Department, Soongsil University, 511 Sangdo-Dong, Dongjak-Gu, Seoul 156-743, Korea; E-Mail: lqsdust@163.com

**Keywords:** electronic mobility aids, sensor fusion, object detection, Bayesian network, context-aware guidance, multimodal information transformation

## Abstract

A wearable guidance system is designed to provide context-dependent guidance messages to blind people while they traverse local pathways. The system is composed of three parts: moving scene analysis, walking context estimation and audio message delivery. The combination of a downward-pointing laser scanner and a camera is used to solve the challenging problem of moving scene analysis. By integrating laser data profiles and image edge profiles, a multimodal profile model is constructed to estimate jointly the ground plane, object locations and object types, by using a Bayesian network. The outputs of the moving scene analysis are further employed to estimate the walking context, which is defined as a fuzzy safety level that is inferred through a fuzzy logic model. Depending on the estimated walking context, the audio messages that best suit the current context are delivered to the user in a flexible manner. The proposed system is tested under various local pathway scenes, and the results confirm its efficiency in assisting blind people to attain autonomous mobility.

## 1. Introduction

According to recent statistics [1], 285 million people worldwide are estimated to be visually impaired worldwide, and among these, 39 million are completely blind. The loss of independent mobility is a large problem for these blind people, and they have to rely on the white cane as their primary mobility tool. However, the white cane has a restricted searching range. Therefore, considerable efforts have been made over the last 20 years to complement white canes with various types of electronic devices to aid mobility. Compared with white canes, these devices can help monitor a wider range of the environment and provide helpful feedback in various modalities. The most critical aspects of developing these electronic mobility aid devices are two-fold: how to sense the environment and how to inform the blind user [2]. In general, the environment can be sensed with various sensors, such as ultrasonic sensors, laser sensors and cameras. Users can be informed via auditory or tactile sense.

In recent years, the camera has gained attention in studies on environment sensing because of its several advantages, such as providing a large sensing area and rich information. A single camera or stereo cameras have been widely used in building electronic mobility aid systems. A single camera is more compact and easier to maintain than stereo cameras are. However, it is difficult to recover depth from a single image. Therefore, to distinguish foreground objects from the background, systems using the single camera have to make strong assumptions about scene geometry and features of appearance. For example, the “NAVI (Navigation Assistance for Visually Impaired)” system [3] uses gray-scale features to discriminate objects from the background. It classifies image pixels into background or objects using a fuzzy neural network. In [4], a color histogram is used to discriminate the ground from the objects. In [5,6], diagonally distributed road boundaries are extracted to find the path area, and then, the objects inside path area are detected by quasi-vertical edges or changes in texture patterns. In [7], by mapping the original image to a top-view plane, objects are detected and classified in the top-view space using the geometric features of object edges. Despite the efforts made to detect objects from a single image without direct depth cues, the appearance and geometry models used in these systems are valid only in limited scenarios.

Compared to a single camera, stereo cameras are more popularly used for building mobility aid systems, because depth can be computed directly from pairs of stereo images. The dense depth map can be used as a valuable asset in object detection and scene interpretation. Some of the systems directly quantize a depth map into a rectangular block representation, and then convert it into tactile vibrations or 3D sound that is perceived by blind users. For instance, the “TVS (Tactile Vision System)” [8] and “Tyflos” system [9–12] convert quantized depth maps into vibrations on a tactile sensor array that is attached to the user's abdomen. The “ENVS (Electron-Neural Vision System)” [13] transforms a vertically divided depth map into electrical pulses that stimulate the user's fingers. In [14,15], a depth map is mapped into a virtual acoustic space.

Instead of mapping the depth map directly to other modalities, some other systems have attempted to improve the resolution of scene interpretation using the depth map. For example, in [16], 3D scene points recovered from the depth map are classified as either in the ground or the object by estimating the ground plane. A polar accumulative grid is then built to represent the scene. In [17], a multi-level surface patch model is built for object representation. In [18], object regions are extracted by segmenting them on a saliency map, and the corresponding depth is computed by using the depth map. Moreover, some systems also include the SLAM algorithm to maintain a local 3D map for object detection, as in [17]. In [19], aerial objects are detected using a local 3D map built from a SLAM algorithm. Although many stereo-vision-based systems have attempted to improve the resolution of scene interpretation, inherent problems still exist in stereo-vision methods. First, the stereo-matching algorithm often fails in texture-less areas, where depth cues are largely unavailable. Second, the accuracy of the depth map is sensitive to illumination and artifacts in the scene. Noise contained in the depth map complicates the identification of low-level objects, such as road curbs. Moreover, the generation of an accurate and dense depth map is still expensive, which reduces the possibility of adding other useful functions with respect to limited computation resources on a portable platform.

Compared with stereo-cameras, laser sensors can produce accurate range data that is not easily affected by environmental conditions. However, this usually requires laser sensors to scan the space to get a full 3D map, which is inconvenient in a walking-guidance task. One such example is the hand-held point-laser device designed by Manduchi, R. [20]. This device requires the user to swing it around in the space for range data collection. Because of this constraint, in some other human navigation systems, laser sensors are used mostly for indoor localization and 2D map building instead of object detection. For example, a combination of a laser scanner and a gyroscope is used in an indoor localization tool [21]. The tool is designed to locate blind users inside a building by matching laser corner features with the corners recorded in a known building map. In [22], a laser scanner and an inertial sensor are fixed on a helmet worn by the user for the purpose of indoor-map building and self-localization. A similar application of a laser scanner is in a wearable multi-sensor system developed in [23], which enables multi-floor indoor mapping and localization. These systems are designed mainly for indoor-map building, and forward-looking laser sensors can only detect high-level objects that are higher than the mounting position.

Moreover, following the recent development of the RGB-D camera, guidance systems using this kind of camera have also emerged [24–28]. The RGB-D camera can obtain both an RGB color map and a depth map of the whole scene in real time. Therefore, it may be used conveniently in both object detection and scene interpretation. However, the RGB-D camera depends on emitted infrared rays to generate a depth map, and in an outdoor environment, the infrared rays can be easily affected by sunlight. Therefore, guidance systems developed using the RGB-D camera can only be used in indoor environments, which limits the range of its use in a mobility aid system.

In this paper, a context-aware audio guidance system is proposed. The contribution of this paper is three-fold. First, a downward-pointing multi-sensor configuration is proposed to cover all types of objects, especially low-level objects that are close to the ground. Second, a multimodal profile model is proposed to interpret the scene at a high resolution. The third contribution is an audio feedback scheme based on the walking context.

## 2. System Overview

The above review of the existing research revealed aspects that need improvement. For environment sensing, the improvement of scene interpretation resolution will help improve users' perceptions of the scene. An ideal scene interpretation resolution would include identifying the location of both the ground each object, as well as the type of each object. However, most existing systems can only interpret the scene at a level of two categories, either the ground plane or the object. They lack the ability to identify one object from the other. For user feedback, with the increase in scene interpretation resolution and context information, there will be an increased demand for delivering highly semantic information to the user. However, the commonly used tactile and virtual sound interfaces have difficulty in meeting this demand. In this paper, we propose a guidance system solution that aims at solving these problems.

The discussion in Section 1 indicates that laser sensors and cameras have properties that may allow them to complement each other. For example, the camera can capture an entire scene with rich intensity data in one frame shot, but it is hard to obtain stable and accurate depth cues from a single image. Although the laser sensor can provide stable and accurate range data, it requires a time-consuming scan to collect sufficient data that covers the whole scene. Therefore, these two types of sensors have the potential to be integrated in order to provide real-time multimodal data for use in the guidance tasks of blind people.

**Figure 1.**
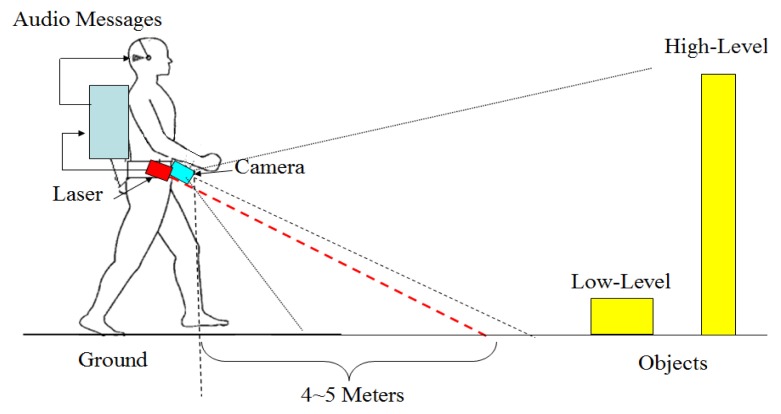
Overview of the system prototype.

Figure 1 shows an illustration of the system prototype. A camera and a laser range finder are fixed with a downward-pointing angle on the waist of a blind user. In this viewing angle, both low and high objects can be captured by the combined sensors. As the user traverses a local environment, two sensors collect environment data in their respective modalities, and the incoming sensor data is further processed on a portable computer, where high-level information regarding scene geometry, objects and walking context is inferred. Finally, the inferred results are forwarded to the user in a flexible manner via audio messages.

The system functions are divided into three parts: moving scene analysis, walking context estimation and audio message generation. The task of moving scene analysis is to detect the ground and objects, while estimating a safe path. Based on the output of moving scene analysis, the walking context estimation evaluates the current walking status as safe, normal or dangerous. Finally, according to the estimated walking context, the audio message generation selects critical information from the moving scene analysis and delivers it to the user at the right moment.

## 3. Moving Scene Analysis

### 3.1. Multimodal Profile Model

In real scene analysis, because of various ground conditions, sensor data noise and unstable sensor motion, large uncertainties exist when separating low-level objects from the ground in the sensor data. Similarly, because of large variances in the shape, size and appearance of objects, many uncertainties also occur when identifying individual objects. To minimize the uncertainties and to obtain the optimal inference of the scene content, a multimodal profile model based on a Bayesian network is proposed. The optimal scene model parameters are inferred by maximizing their joint probability distributions.

**Figure 2.**
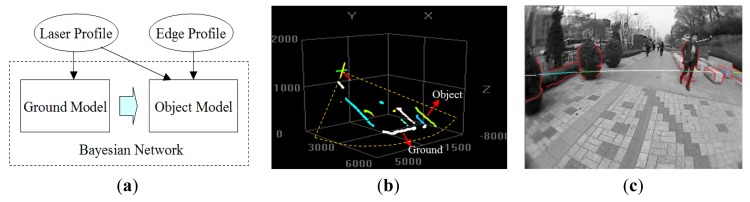
Overview of multimodal profile model. (**a**) General diagram; (**b**) laser range data; (**c**) fusion of the image and laser data.

**Figure 3.**
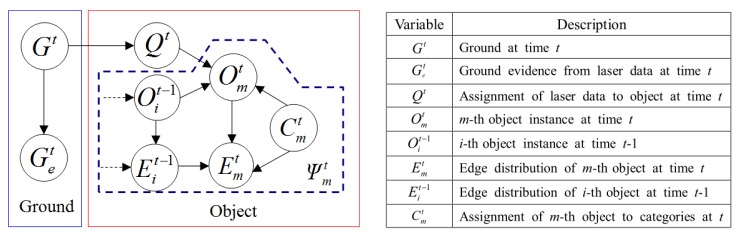
Multimodal profile model.

As illustrated in Figure 2a, the proposed multimodal profile model is composed of two parts: the ground model and the object model. In this model framework, the data from two sensor modalities play different roles. As shown in Figure 2b, the laser profile data are used as the main cues for building the ground model. However, the features of these depth profiles are too limited to identify object types. As shown in Figure 2c, edge profiles are extracted from the image and combined with associated laser profiles to form a multimodal representation of the object, which is used in building the object model. The ground model and the object model are unified in a Bayesian network, so that the optimal model parameters can be inferred to minimize uncertainties in this process. Figure 3 illustrates the multimodal profile model in the form of a Bayesian network. Using this model, the moving scene analysis becomes a problem of maximizing the joint probability distribution of all of the random variables involved. This joint probability distribution can be derived as shown in Equations (1)–(4).

(1) $$P(G^{t},G_{e}^{t},Q^{t},\Psi^{t})=P(G^{t},G_{e}^{t},Q^{t})P(\Psi^{t}|G^{t},G_{e}^{t},Q^{t})$$

(2) $$\begin{array}{ll} P(G^{t},G_{e}^{t},Q^{t}) & =\;P(G^{t})P(G_{e}^{t}|G^{t})P(Q^{t}|G^{t},G_{e}^{t})\\ & =P(G^{t})P(G_{e}^{t}|G^{t})P(Q^{t}|G^{t}) \end{array}$$

(3) $$P(\Psi^{t}|G^{t},G_{e}^{t},Q^{t})=\sum_{m=1}^{n}P(\Psi_{m}^{t}|Q^{t})$$

(4) $$\begin{array}{ll} P(\Psi_{m}^{t}|Q^{t}) & =P(O_{i}^{t-1})\;P(O_{m}^{t}|Q^{t})P(O_{m}^{t}|O_{i}^{t-1})\\ & \cdot P(E_{i}^{t-1}|O_{i}^{t-1})\;P(E_{m}^{t}|O_{m}^{t})P(E_{m}^{t}|E_{i}^{t-1})P(C_{m}^{t}|E_{m}^{t},O_{m}^{t}) \end{array}$$

In Equation (1), $P(G^{t},G_{e}^{t},Q^{t})$ is the joint probability of ground location and laser points labeling, and $P(\Psi^{t}|G^{t},G_{e}^{t},Q^{t})$ denotes the joint probability of all objects Ψ*^t^* given ground location and laser data labeling. In Equation (2), $P(G^{t})P(G_{e}^{t}|G^{t})\propto P(G^{t}|G_{e}^{t})$ is the posterior probability of ground *G^t^* by observing ground evidence $G_{e}^{t}$ , and $P(Q^{t}|G^{t},G_{e}^{t})$ gives the probability of assigning laser data to the object given estimated ground *G^t^*. Because *Q^t^* and $G_{e}^{t}$ are conditional independent given *G^t^*, $P(Q^{t}|G^{t},G_{e}^{t})$ is equivalent to P(*Q^t^* | *G^t^*). In Equation (3), the joint probability of all objects is approximated as the total probability of each object instance. Here, we assume that the probability of each individual object is independent.

In Equation (4), $P(O_{m}^{t}|Q^{t})$ is the probability of the *m*-th object instance given object laser point labeling *Q^t^*, $P(O_{m}^{t}|O_{i}^{t-1})\;$ is the temporal correlation of the *m*-th object instance, $P(E_{m}^{t}|O_{m}^{t})$ indicates the fusion of the *m*-th object's laser profile and the edge profile in frame *t*, $P(E_{m}^{t}|E_{i}^{t-1})$ is the temporal correlation of the *m*-th object's edge distribution and $P(C_{m}^{t}|E_{m}^{t},O_{m}^{t})$ indicates the object's type assignment probability given the *m*-th object's multimodal profile. In the following sections, each probability distribution in the model will be defined, and the methods used to find their maximum probability values will be discussed.

### 3.2. Ground Estimation

**Figure 4.**
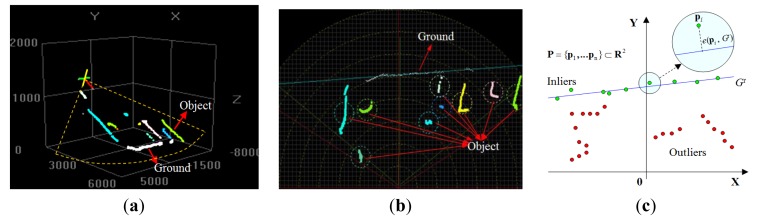
Ground estimation in laser scanning frame. (**a**) Laser data in 3D frame; (**b**) laser data in 2D scanning frame; (**c**) ground model definition.

As shown in Figure 4a, because the laser scanner points downward at a fixed angle, some of the laser points are reflected from the ground, and the others are reflected from the objects. Figure 4b shows laser data observed in a 2D scanning frame. Assuming that the ground plane has a low curvature, a linear model is used to approximate the ground plane geometry. As shown in Figure 4c, in the laser's scanning frame, the ground at time *t* is defined as *G^t^*: *g_1_*x + *g_2_*y + *g_3_* = 0. To find the *G^t^* that is the most probable to be the real ground G^t*^, a MAP (Maximum-a-Posteriori) estimation of G^t*^ is searched as $\underset{G^{t}}{\arg\max}P(G^{t}|G_{e}^{t})$. Using the Bayesian rule, the posterior probability $P(G^{t}|G_{e}^{t})$ can be derived as $P(G^{t}|G_{e}^{t})\propto P(G^{t})P(G_{e}^{t}|G^{t})$, where P(*G^t^*) is the prior probability of *G^t^* and $P(G_{e}^{t}|G^{t})$ is the likelihood of a given *G^t^* by observing ground evidence $G_{e}^{t}$ .

Ground evidence $G_{e}^{t}$ is measured through the point-wise error *e_i_*(**p***_i_*, *G^t^*), which is defined as the Euclidean distance between the laser point and the hypothesized line model. As illustrated in Figure 4c, if *G^t^* is the ground, then the laser points on the ground can be regarded as the “inliers” of this *G^t^*, and the laser points on objects can be regarded as the “outliers” of *G^t^*. With this observation, the probability distribution of *e_i_*(**p***_i_*, *G^t^*) is modeled as a mixture of two parts, as in Equation (5). The inlier error is modeled using a Gaussian distribution, and the outlier error is modeled as a uniform distribution. The two parts are combined by using a radio parameter *r*. Assuming that *e_i_*(**p***_i_*, *G^t^*) at each point is independent from the others, $P(G_{e}^{t}|G^{t})$ can be defined as shown in Equation (6).

(5) $$P(e_{i})=r\frac{1}{\sqrt{2\pi \sigma^{2}}}\exp(-\frac{e_{i}^{2}}{2\sigma^{2}})+(1-r)\frac{1}{v}$$

(6) $$P(G_{e}^{t}|G^{t})=\prod_{i=1}^{n}P(e_{i})$$

**Figure 5.**
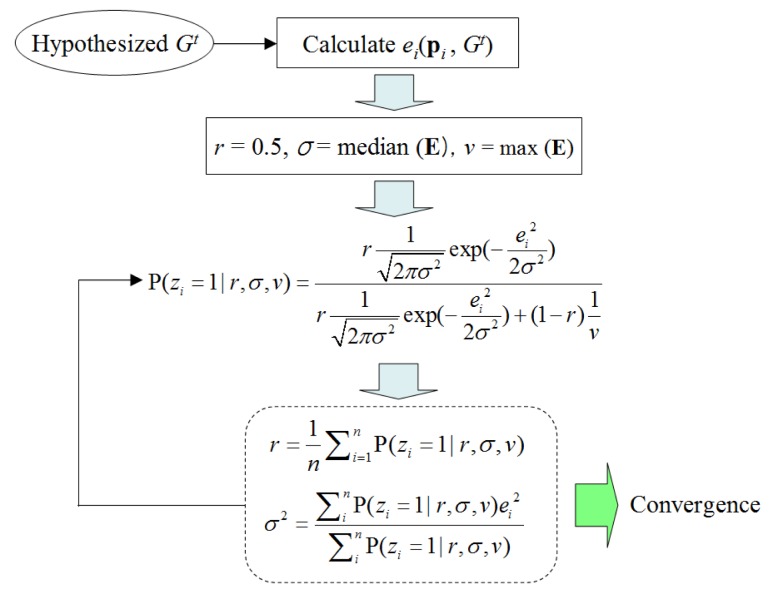
EM procedure for fitting *P*(*e_i_*).

To calculate P(*e_i_*) using the mixture probability distribution defined in Equation (5), three parameters (*r*, *σ*, *v*) must be determined. Given a set of laser point data, the EM (Expectation-Maximization) algorithm is used here to fit (*r*, *σ*, *v*). As Figure 5 shows, for a given model hypothesis *G^t^*, the set of all point errors **E** = {*e*_1_,*e*_2_,…*e*_n_} is used to initialize (*r*, *σ*, *v*), which is then updated iteratively by calculating the probability of each laser point belonging to the ground. Here, a set of indicator variables *z_i_* is introduced; *z_i_* = 1 indicates that the *i*-th data point is from the ground, and *z_i_* = 0 means that it is from the object. Here, *z_i_* is treated as missing data, and its probability values are updated iteratively together with (*r*, *σ*, *v*) in order to approach the best values. This iterative procedure is repeated until convergence.

A random sampling scheme is used to form the ground model hypothesis. Two laser points are randomly selected in the laser data frame to give a hypothesized line model *G^t^*. For each *G^t^*, $P(G_{e}^{t}|G^{t})$ is calculated using P(*e_i_*), the pdf (Probability Density Function) of which can be fitted through the above EM procedure. Finally, among all hypothesized *G^t^*, the one with the maximum $P(G_{e}^{t}|G^{t})$ is chosen as the ML (Maximum Likelihood) estimation of the real ground.

**Figure 6.**
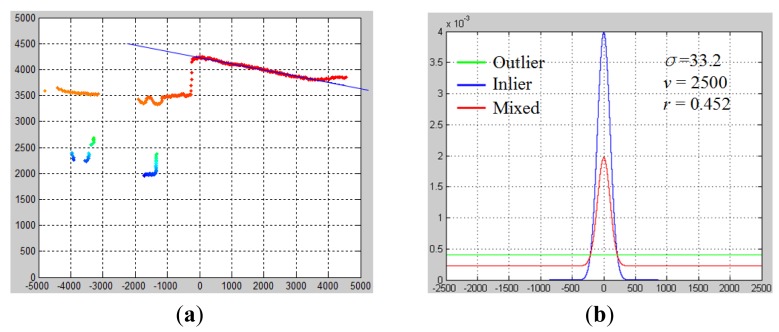
An example of the ground estimation result. (**a**) Ground probability map; (**b**) fitted error distribution.

The results of ground estimation are shown in Figure 6. Figure 6a shows the best ground line model G^t*^ among all hypothesized *G^t^*, and Figure 6b shows the fitted error probability distribution with respect to this G^t*^. Moreover, when EM converges, a ground probability map can be obtained using P(*z_i_* = 1), as is denoted by the heat color indicated in Figure 6a. This ground probability map is a valuable cue for labeling laser data points as in the ground or in objects.

To obtain a MAP estimation of the ground, instead of using random sampling, a guided sampling scheme is used to generate hypothesized *G^t^*. Here, each data point is assigned a sampling weight to denote its importance in the sampling process. An initial sampling weight is assigned using a 2D Gaussian distribution, as in Equation (7), where (*x*, *y*) is a point in the laser's 2D scanning frame and (*x_0_*, *y_0_*) is the ground center position obtained from prior knowledge. For the frames following the initial frame, P(*G^t^*) is modeled using the posterior probability of the ground model at time *t*−1. The final prior probability P(*G^t^*) is defined as in Equation (8). In the initial frame, P(*G^t^*) is determined by the initial sampling weight of two laser points. In the following frames, P(*G^t^*) is determined by the posterior probability of the ground model in the previous frame.

(7) $$P(x,y)=A\exp(-(\frac{{\left(x-x_{0}\right)}^{2}}{2\sigma_{x}^{2}}+\frac{{\left(y-y_{0}\right)}^{2}}{2\sigma_{y}^{2}}))$$

(8) $$P(G^{t})=\{\begin{array}{ll} P(x_{1},y_{1})*P(x_{2},y_{2}) & t=0\\ P(G^{t-1}|G_{e}^{t-1}) & t>0 \end{array}$$

### 3.3. Object Detection

#### 3.3.1. Object Probability Map Labeling

**Figure 7.**
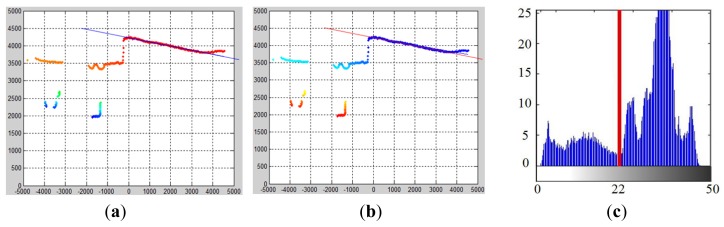
Object probability map. (**a**) Ground probability map; (**b**) object probability map; (**c**) optimal labeling threshold.

After the best ground model G^t^* is found, P(*z_i_* = 0) gives the probability of each laser point belonging to the object. By using P(*z_i_* = 0), an object probability map can be obtained, which is a reversed version of the ground probability map, as shown in Figure 7b. Using this object probability map, the problem of object detection can be modeled as seeking the optimal labeling of laser points as belonging to objects or the ground.

In the multimodal profile model, *Q^t^* is used to denote such a labeling. Searching for the optimal labeling emerges as another MAP estimation problem as: $Q^{t*}=\underset{Q^{t}}{\arg\max}P(Q^{t}|G^{t})$ . By applying the Bayesian rule, the posterior probability P(*Q^t^* | *G^t^*) can be written as P(*Q^t^* | *G^t^*) ∝ P(*Q^t^*) P(*G^t^* | *Q^t^*). Assuming each labeling is equally likely to be Q^t^*, P(*Q^t^*) can be omitted. The P(*G^t^* | *Q^t^*) is measured using a between-class variance $\sigma_{\text{og}}^{2}$. To define this $\sigma_{\text{og}}^{2}$ , a histogram of the object probability map is built as shown in Figure 7c. The horizontal axis is −log(P(*z_i_* = 0)) ranging from zero to 50, and the vertical axis is the number of laser points that fall in each bin. Based on this minus-log-probability histogram, the hypothesized labeling *Q^t^* is formed by choosing one of the bins as a threshold to divide the laser points into either the object or the ground class, and the between-class variance $\sigma_{\text{og}}^{2}$ can be calculated as in Equation (9), where *r*_obj_ and *r*_gnd_ are the ratio of the object laser points and the ground laser points and *ì*_obj_ and *ì*_gnd_ are the mean minus-log-probability of the object class and ground class. Finally, the *Q^t^* with the largest $\sigma_{\text{og}}^{2}$ is selected as the optimal labeling Q^t*^. In Figure 7c, the bin with the value “22” is selected as the best threshold. The minus-log-probability of 22 corresponds to a probability value of 0.82. Therefore, in the object probability map, a laser point with a probability value larger than or equal to 0.82 is labeled as belonging to the object.

(9) $$\sigma_{\text{og}}^{2}=r_{\text{obj}}r_{\text{gnd}}{\left(\mu_{\text{obj}}-\mu_{\text{gnd}}\right)}^{2}$$

#### 3.3.2. Laser Profile Clustering

Object detection at a high resolution is a highly uncertain process. A MAP estimation of P(*Q^t^* | *G^t^*) can reduce uncertainties when separating object laser points from ground laser points. In order to handle uncertainties when separating individual objects from each other, the maximum value of $P(O_{m}^{t}|Q^{t})$ is to be searched. Assuming that the object generally has a smooth surface profile, then $P(O_{m}^{t}|Q^{t})$ can be measured according to the degree of smoothness observed in the laser data. This turns into a laser profile clustering problem. Here, we solve this problem based on a mean shift clustering scheme.

Mean shift clustering can be considered as a nonparametric kernel density estimation [29,30], where an unknown density function f(*x*) of the data is estimated via a kernel density function $\hat{f(x)}$ . The local maxima of the estimated kernel density function then yields cluster centers of the data. A general form of the kernel density function is given by Equation (10), where *K* is the kernel function and *h* is the kernel size.

(10) $$\hat{f(x)}=\frac{1}{nh^{d}}\sum_{i=1}^{n}K(\frac{x-x_{i}}{h})$$

Based on this kernel density estimation scheme, the kernel density function is defined to determine the smooth laser clusters. Now, given a set of object laser points **P** = {**p**_1_,…**p**_n_}, the goal is to cluster a smooth profile in this set of laser points. Here, a local smooth likelihood function S*_i_*(**x**) is defined in a neighborhood of **p***_i_* to estimate the probability that a point **x** is located on a local object profile in this neighborhood. To define S*_i_*(**x**), a combination of two kernels is used.

**Figure 8.**
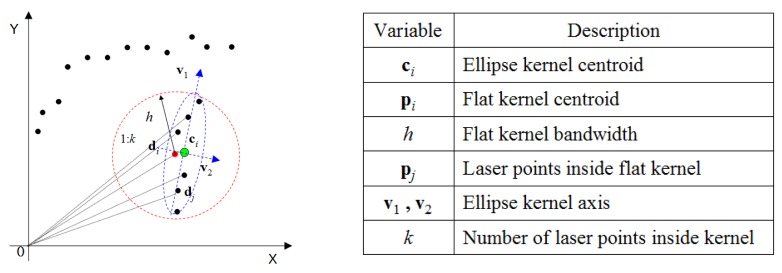
Definition of circular and ellipse kernels.

As is shown in Figure 8, the flat circular kernel determines the scale of the neighborhood to be evaluated, depending on kernel size *h*. All laser points that are included in the circular kernel are used to calculate an ellipse kernel **E***_i_* by applying PCA (principal component analysis) to the involved laser points, as in Equation (11), where **c***_i_* is the centroid and **Σ***_i_* is a covariance matrix with two eigenvectors **v**_1_ and **v**_2_. Because **v**_2_ is the direction in which the smallest depth variance occurs, its orthonormal vector **v**_1_ indicates the most probable position of a local object profile in this neighborhood.

(11) $$E_{i}(\text{x})=\{\text{x}:{\left(x-{\text{c}}_{i}\right)}^{\text{T}}\Sigma_{i}^{-1}(x-c_{i})\leq 1\}\;\;$$

(12) $$S_{\text{i}}(x)=N_{\text{i}}(x,c_{i},\Sigma_{i})[h^{2}-{\left[(x-c_{i})\cdot v_{2i}\right]}^{2}]$$

**Figure 9.**
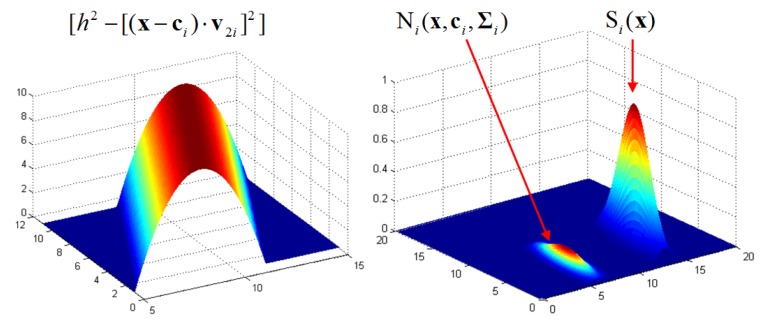
Local smooth likelihood function.

The smoothness likelihood S*_i_*(**x**) of this local neighborhood is then evaluated with respect to **v**_1_ as in Equation (12). The squared distance from a position **x** to the possible object profile location **v**_1_ is used to measure the smoothness, which reflects the probability of a position **x** ∈ **R**^2^ located on this profile. A 2D anisotropic Gaussian weighting function is used to diminish the possibility of distant points on the local profile. For each sampled laser point **p***_i_*, a corresponding *S_i_*(**x**) can be calculated, which is illustrated in Figure 9. By accumulating all of the *S_i_*(**x**) defined at each laser point in the neighborhood, a complete kernel density function $\hat{f(x)}$ can be obtained as in Equation (13), where *K* is a normalizing factor. This kernel density function $\hat{f(x)}$ is used to approximate the unknown object density $P(O_{m}^{t}|Q^{t})$ .

(13) $$P(O_{m}^{t}|Q^{t})\propto \hat{f(x)}=\frac{1}{K}\sum_{i=1}^{n}S_{i}(x)$$

**Figure 10.**
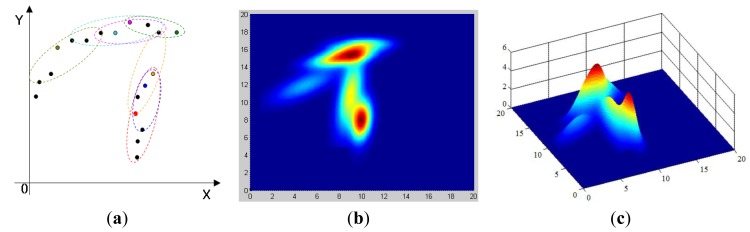
Accumulated smooth likelihood function. (**a**) Ellipse kernel in the laser frame; (**b**) accumulated smooth likelihood function in the 2D view and 3D view.

As shown in Figure 10, the local smoothness likelihood S*_i_*(**x**) defined at each ellipse kernel is accumulated to approximate the unknown pdf of $P(O_{m}^{t}|Q^{t})$. Ellipse kernels that have similar positions and orientations will accumulate a higher probability vote on the positions involved, which reflects that they are more likely to locate on a smooth profile. The locations that get the largest vote are the cluster centers that indicate the existence of an object profile.

Using the defined $\hat{f(x)}$ , the optimal segmentation of objects can be found by searching for local maxima in $\hat{f(x)}$. Here, a mean shift scheme based on gradient ascent maximization is used, as is defined in Equation (14). An iteration procedure from a sample point **p***_i_* converges if the mean shift vector is less than the given threshold.

(14) $$\begin{array}{c} \begin{array}{cc} p_{i}^{0}=p_{i}, & p_{i}^{k+1}=p_{i}^{k}-m_{i}^{k} \end{array}\\ m_{i}^{k}=\frac{\sum_{j=1}^{n}N_{j}(p_{i}^{k}-c_{j})\cdot [(p_{i}^{k}-c_{j})\cdot n_{j}]\cdot n_{j}}{\sum_{j=1}^{n}N_{j}(p_{i}^{k}-c_{j})} \end{array}$$

The above mean shift process is applied to every laser point in order to allow each one to converge to the nearest local maxima. After merging local maxima that are sufficiently close to one another, the final cluster centers can be determined. Each laser point is assigned to the cluster center to which it converges, and each laser profile cluster represents an individual object.

### 3.4. Multi-Category Object Classification

#### 3.4.1. Pixel-Level Fusion of Laser and Image Data

Although laser data are efficient for ground and object detection, the depth profile they provide is still limited with regard to identifying object categories. Therefore, the edge profiles from images are used to supplement laser profiles in order to classify objects into multiple categories. To combine the edge profiles with the laser profiles, sensor calibration is performed to map laser profiles into the image domain.

**Figure 11.**
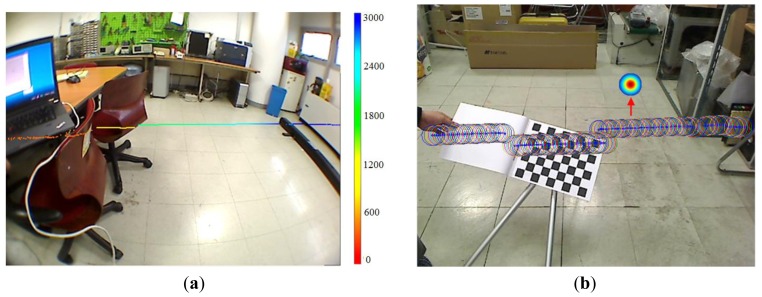
Mapping laser points into the image frame. (**a**) Pixel-level fusion of laser and image data; (**b**) fusion uncertainties on the image plane.

We use the method proposed in [31] to calibrate the laser sensor with the camera. After calibration, a mapping relationship is determined in order to map a laser point **P**_l_ from the laser's coordinate system into the point **P**_i_ in the image plane, as in Equation (15), where <**Φ Δ**> gives the mapping relationship between the laser and camera coordinates, **K** is the camera's intrinsic matrix and **d** is the non-linear distortion parameters. The example of a mapping result is shown in Figure 11a.

(15) $$P_{\text{i}}=f(P_{\text{l}},\Phi^{-1},\Delta ,\;K,\;d)$$

In Equation (15), the set of calibration parameters <**Φ**, **Δ**, **K**, **d**> may contain errors. Therefore, uncertainties exist when using this parameter set to map **P**_l_ to **P**_i_. Here, we want to estimate the uncertainty of a non-linear mapping function f(**ξ**), based on the uncertainty of its mapping parameters ξ = (**Φ**, **Δ**, **K**, **d**). By using the non-linear covariance propagation theorem [32], the covariance matrix of f(ξ) can be approximated by Equation (16), where Σ_ξ_is the covariance matrix of calibration parameter vector ξ, and **J_f_** is the Jacobian matrix of f(ξ), evaluated at **ξ̄**.

(16) $$\sum_{\text{li}}=J_{\text{f}}\sum_{\xi }J_{\text{f}}^{\text{T}}$$

Given one laser point **P_l_**, a 2 × 16 Jacobian matrix **J_f_** can be calculated with respect to the 16 calibration parameters. Thus, Equation (16) finally produces a 2 × 2 covariance matrix $\sum_{\text{li}}=\mathrm{diag}(\sigma_{\text{u}}^{2},\sigma_{\text{v}}^{2})$ , which gives the deviation of the coordinates of **P**_i_ mapping from **P**_l_. Figure 11b shows this pixel-level fusion uncertainty. The mapping position on the image is shown in blue dots, and the uncertainty of each mapped position is illustrated using an ellipse centered at the mapping position. The major and minor axes of the uncertainty ellipse are spanned by 3*σ*_u_ and 3*σ*_v_, representing 95% of the probability that the mapping position lies in this ellipse.

#### 3.4.2. Profile-Level Fusion of Laser and Image Data

Based on the pixel-level fusion of the laser points and image pixels, we can further move to the profile-level fusion of the laser profile and edge profile. This multimodal profile fusion is represented as $P(E_{m}^{t}|O_{m}^{t})$ in the multimodal profile model.

**Figure 12.**
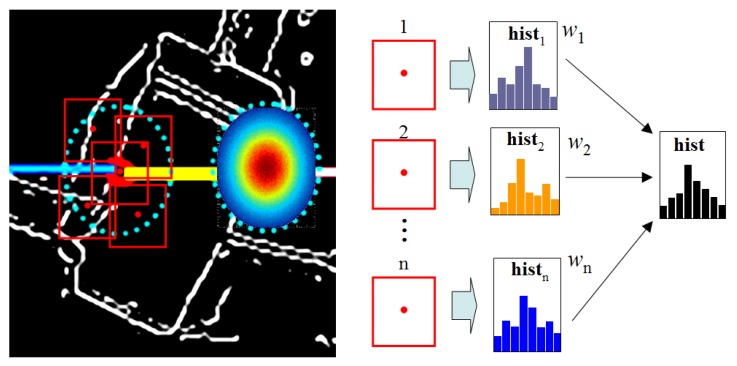
Profile-level fusion of laser and image data.

Figure 12 illustrates the modeling of $P(E_{m}^{t}|I_{m}^{t})$ in the form of a weighted edge-orientation histogram. In general, object laser profiles are supposed to break up at object boundaries. Therefore, if edge pixels are traced from these break-points on the image, useful local features concerning object shape can be obtained. In Figure 12, the image on the left side shows object laser profiles mapping onto the edge map, where Σ_li_ at each break point specifies an ellipse neighborhood, within which the mapping uncertainties can be modeled as a 2D Gaussian distribution.

As Figure 12 shows, inside this ellipse neighborhood, each sampled point specifies a rectangular window. For each rectangular window, a chain-code histogram is built to represent the edge orientation distribution around this neighborhood. Finally, all histograms are combined into one histogram, as in Equation (17), where **h***_i_* is a vector containing eight bins and *w_i_* is a weight that is determined by the 2D Gaussian distribution that is used to model pixel-level fusion uncertainty.

(17) $$H=\sum_{i=1}^{n}w_{i}h_{i}$$

**Figure 13.**
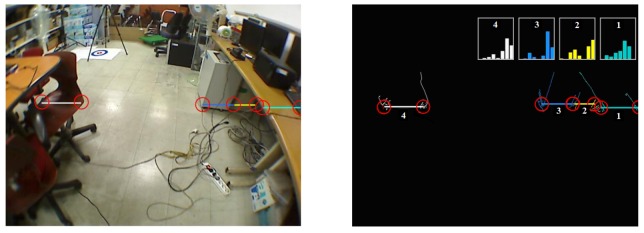
An example of the weighted chain-code histogram.

The weighted chain-code histogram can work effectively as a local shape descriptor. For objects with quasi-vertical side-profiles, a dual-peak pattern at Bin 2 and Bin 6 can be observed in the chain-code histogram, whereas for objects with lateral side-profiles, a dual-peak pattern at Bin 3 and Bin 7 can be observed. Therefore, the chain-code histogram can provide useful shape cues with which to identify vertical objects with lateral objects. An example of chain-code histogram extraction in a real scene is shown in Figure 13. The laser profile and its associated edge profile constitute a multimodal profile representation of the object.

#### 3.4.3. Temporal Correlation of Multimodal Profile Model

To further reduce uncertainties in moving scene analysis, temporal correlation cues across adjacent data frames are included in the multimodal profile model. The temporal correlation cues are represented as $P(O_{m}^{t}|O_{i}^{t-1})$ and $P(E_{m}^{t}|E_{i}^{t-1})$ in the multimodal profile model.

**Figure 14.**
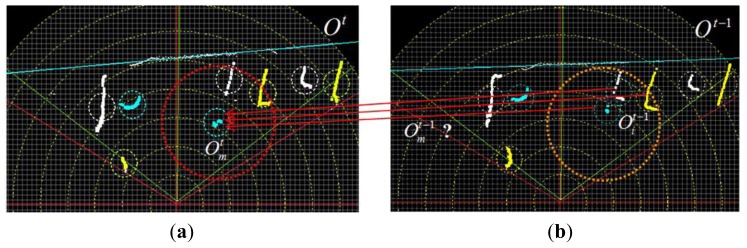
Laser profile association. (**a**) Laser profile in time *t*; (**b**) laser profile in time *t−*1*.*

Here, a Bayesian tracker based on data association is proposed. As shown in Figure 14, there is a collection of *l* object profiles *O^t^* at time *t* and a set of *k* object profiles *O^t^*^−1^ at time *t*−1. For one object profile $O_{m}^{t}$ in *O^t^*, decisions should be made regarding which object profile in *O^t^*^−1^ corresponds to $O_{m}^{t-1}$ or whether no object profile corresponds to $O_{m}^{t-1}$ , when $O_{m}^{t}$ is a newly-emerging object. The posterior probability of each object profile in *O^t^*^−1^ being $O_{m}^{t-1}$ is evaluated as in Equation (18), where $P(O_{i}^{t-1})$ is the prior probability that each object profile in *O^t^*^−1^ is $O_{m}^{t-1}$ , and $P(O_{i}^{t-1}|O_{m}^{t})$ is the likelihood of each object profile in *O^t^*^−1^, given $O_{m}^{t}$ as an on-line model.

(18) $$P(O_{m}^{t}|O_{i}^{t-1})\propto P(O_{m}^{t})P(O_{i}^{t-1}|O_{m}^{t})$$

The prior probability term $P(O_{m}^{t})$ is modeled as a 2D isotropic Gaussian distribution N*_m_*(**x**, **u***_m_*, **Σ**), where **x** is a position in the laser's scanning frame and **u***_m_* is the centroid of laser profile $O_{m}^{t}$ . The variance σ*_x_*, σ*_y_* is specified using the largest movement *T* of an object laser profile between adjacent frames. The likelihood of $P(O_{i}^{t-1}|O_{m}^{t})$ is calculated using the ICP (iterative closest point) algorithm. By applying ICP matching between model profile $O_{m}^{t}$ and each target profile $O_{i}^{t-1}$ , an optimal transformation <**R***_i_*, **t***_i_*> can be obtained. In addition, a point-wise error can be calculated as $e_{j}=\left|\overset{\wedge }{{\text{x}}_{j}}-Rx_{j}-t\right|$ . Here, a threshold *τ* is applied on each *e_j_* and the point **x***_j_*, whose transformed error *e_j_* is smaller than τ, is defined as an inlier, and the remaining points are all regarded as outliers. The number of inliers is used to define the likelihood, as in Equation (19), where *N*_inlier_ is the number of inliers in the target profile, *S*_M_ is the number of points in the model profile and *S*_T_ is the number of points in the target profile.

(19) $$P(O_{i}^{t-1}|O_{m}^{t})=\frac{N_{\text{inlier}}}{\max(S_{\text{M}},S_{\text{T}})}$$

By calculating the posterior probability of each target profile $O_{i}^{t-1}$ as $O_{m}^{t-1}$ , the target profile with the highest posterior is selected as $O_{m}^{t-1}$ . Additionally, if none of the candidates has a posterior probability higher than the minimum threshold, then $O_{m}^{t-1}$ is assumed to not exist, and $O_{m}^{t}$ is deemed the newly emerging object profile. When $P(O_{m}^{t}|O_{i}^{t-1})$ reaches the maximum by finding the $O_{m}^{t-1}$ , $P(E_{m}^{t}|E_{i}^{t-1})$ can also be regarded as reaching the maximum with $P(E_{m}^{t}|E_{m}^{t-1})$ , which is calculated as in Equation (20). An accumulated chain-code histogram can be built by propagating the chain-code histogram extracted around the corresponding laser profiles across frames.

(20) $$P(E_{m}^{t}|E_{m}^{t-1})\propto \eta P(E_{m}^{t})+(1-\eta )P(E_{m}^{t-1})$$

The accumulated chain-code histogram is more stable than the one built from the single frame is. When the laser scanner moves forward, the laser profile will project on different parts of one object. In real situations, some parts of this object might suffer from partial occlusion, whereas other parts might not. The accumulated chain-code histogram incorporates the local edge distribution from different local parts of the same object, thus greatly strengthening the histogram's tolerance to partial occlusion.

#### 3.4.4. Object Classification

**Figure 15.**
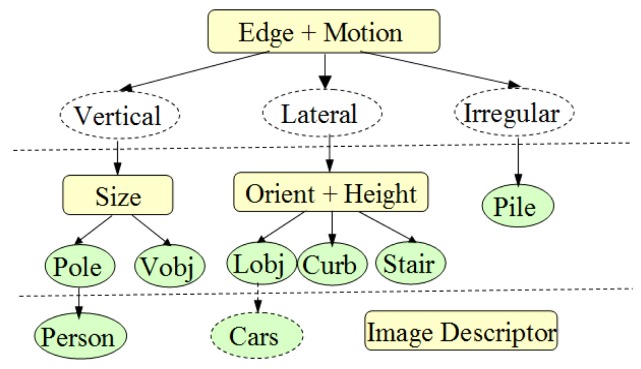
Three-layer structure for object classification.

The chain-code histogram provides shape features from edge profiles. Size, height, orientation and vertical motion are extracted from laser profiles to constitute another four-bin histogram after normalization. Finally, a multimodal profile histogram can be obtained for object classification. A three-layer classification structure is proposed, as shown in Figure 15.

At the first level, three object types are defined. “Vertical Object (Vobj)” have quasi-vertical edges on their side-profiles, such as poles, pedestrians, doors and trees. “Lateral objects (Lobj)” have diagonally distributed edges on their side profiles, such as road curbs, side walls and guardrails. “Irregular objects” do not have regular shapes, such as bushes or piles of stones, and are addressed as “pile” in our system. Edge profiles are used as major features to classify objects in the first level, because it is more stable than the motion feature is, and it does not require the relative movement between the laser sensor and the object. However, if there is no sufficient edge profile that can be extracted around this object, then the motion feature from the laser profile is referred to in the classification process.

At the second level, objects are further classified into more specific categories by using features from the laser profile. “Poles” are vertical objects that have thin cross-sections, usually within a width of 30 cm. “Curb” usually has a lower height and quasi-horizontal profile orientation in the laser data domain. Stairs can also be identified as a specific object out of the “lateral object” group. After fitting the laser profile into a poly-line model, the number of poly-line segments is used as a criterion to identify stairs.

At the third level, a sophisticated image descriptor, such as the HOG (Histogram of Oriented Gradient) [33], is used to identify more specific object types from their “parent-categories”. One of the benefits of this layered structure is that the recognition of more complex object types can be guided by the position of their parent-object instances. The location of their parent-object instance provides an ROI (Region of Interest) that can help reduce the recognition cost of complex object types. Here, in the current stage of our system, only pedestrian recognition is applied at the third level. A pre-trained HOG descriptor is used to match the ROI where the “pole” object is detected. If the matching score is higher than the pre-defined threshold, then the “person” is identified in the “pole” category.

**Figure 16.**
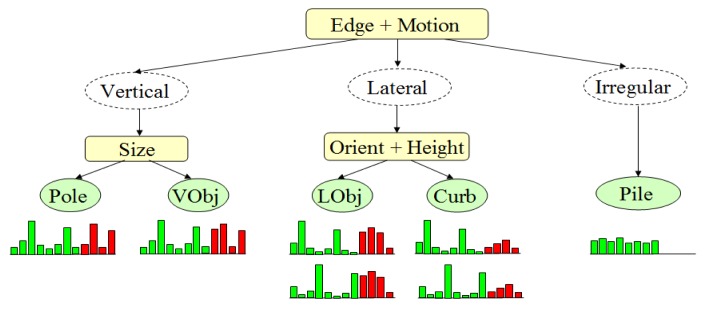
Object category prototype.

In order to perform the classification using the above three-layered approach, the category prototypes of the defined object categories must be built from training data. In brief, after the collection of the training data concerning all object categories, the multimodal profile histogram is extracted for each object in the training data set, and then each multimodal profile histogram is treated as a 12-dimensional feature vector. K-means clustering is then performed in this 12-dimensional feature space to cluster the feature vectors into seven clusters. The cluster center is used as the object category prototype, as shown in Figure 16.

In the multimodal profile model, the probability of assigning an object type to a detected object is described by $P(C_{m}^{t}|O_{m}^{t},E_{m}^{t})$ . According to the Bayesian rule, this probability can be derived as in Equation (21), where $P(O_{m}^{t},E_{m}^{t}|C_{m}^{t})$ is the likelihood of given object prototype *C*, and $P(C_{m}^{t})$ is a prior probability term. In general, it is assumed that all of the object categories are equally likely to appear on the road; therefore, $P(C_{m}^{t})$ can be omitted. The posterior probability is dependent on the likelihood term, $P(O_{m}^{t},E_{m}^{t}|C^{t})$ .

(21) $$P(C_{m}^{t}|O_{m}^{t},E_{m}^{t})=\frac{P(O_{m}^{t},E_{m}^{t}|C_{m}^{t})P(C_{m}^{t})}{P(O_{m}^{t},E_{m}^{t})}$$

(22) $$\begin{array}{l} P(C^{t}=i|H_{m}^{t})=Z{\left(H_{m}^{t}\right)}^{-1}\exp(-d_{i}(H_{m}^{t}))\\ Z(H_{m}^{t})=\sum_{i=1}^{K}\exp(-d_{i}(H_{m}^{t})) \end{array}$$

The likelihood is modeled using the Gibbs distribution, as in Equation (22), where $d_{i}(H_{\text{m}}^{\text{t}})$ represents the histogram distance from a measured multimodal profile histogram $H_{m}^{t}$ to the *i-*th object category prototype histogram, and $Z(H_{m}^{t})$ is a normalization factor. To calculate the histogram distance $d_{i}(H_{m}^{t})$ between a measured histogram and a prototype histogram, the *χ*^2^ distance measurement is used as in Equation (23), where **h_0_** is the prototype histogram and **h***_m_* is the measured histogram. Parameter ***i*** represents the *i-*th histogram bin.

(23) $$\chi^{2}(h_{m},h_{0})=\sum_{i}\frac{{\left(h_{m}[i]-h_{0}[i]\right)}^{2}}{h_{m}[i]+h_{0}[i]}$$

## 4. Audio Message Generation

### 4.1. Walking Context Estimation

**Figure 17.**
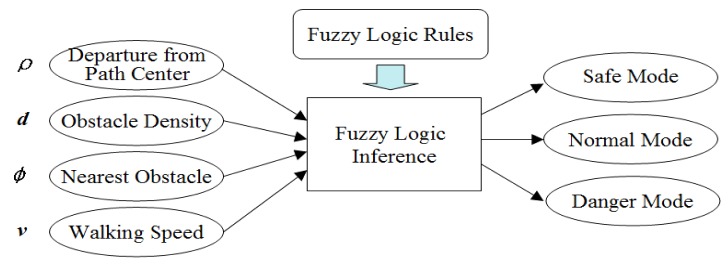
Fuzzy logic model for walking context estimation.

**Figure 18.**
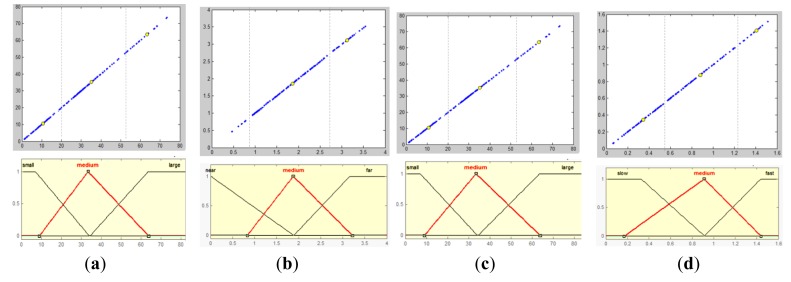
Fuzzification of walking context factors. (**a**) Fuzzification of *ñ*; (**b**) fuzzification of *d*; (**c**) fuzzification of *ö*; (**d**) fuzzification of *v*.

The walking context is defined as the safety level of the walking condition. A fuzzy logic inference model is built to evaluate this safety level, based on several factors defined in the output of the moving scene analysis, as illustrated in Figure 17. To define the membership functions of four input variables, sample data of these four variables are collected from various scenes. By examining the sampled data distribution in their respective domains, data clusters are localized and used to define fuzzy sets, as shown in Figure 18.

The output variable of the walking context is defined as the degree of safety. It is denoted as ***c*** and measured as a percentage value. Because no direct data is available to calculate this value, the intuition and experience of a normal person is used to define its membership function. For each sampled scenario with a set of fuzzy values of four input variables, we let the users decide the degree of safety in this scenario by using their intuition and experience. With a set of fuzzy values of four input variables on one side and the user's answers on the safety degree on the other side, the parameters of a piece-wise linear function are learned from these data.

To perform fuzzy inference, a set of fuzzy rules is also needed. Basically, fuzzy rules take the form of “If…then…” in correlating a set of fuzzy values of input variables to a unique fuzzy value of the output variable. For example, “If ρ is low, and *d* is far, and φ is small and *v* is low, then the walking context *c* is safe”. The fuzzy rules used in our fuzzy logic model are derived from the user-labeled data when the membership function of output variable *c* is learned. Therefore, these fuzzy rules reflect normal human decisions in a walking context under different situations. A list of the fuzzy rules is shown in Table S1, and a fuzzy inference process is illustrated in Figure S1. The result of all fuzzy inference processes can be represented as a solution space shown in Figure S2.

### 4.2. Message Definition

The message structure is defined based on the type of information it expresses. The information that should be delivered to the user is divided into four basic sets, as shown in Table 1. The words in brackets are template words that can be changed according to the result of the detection.

**Figure 19.**
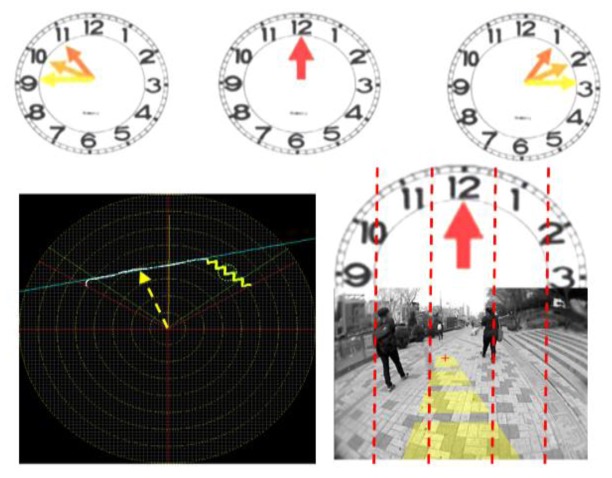
Definition of clock-face direction.

In the safe-direction message set, clock-face directions are used to give messages about direction, as shown in Figure 19. The laser's scanning plane is shown as a big clock-face, in which two leftward directions are specified as 10 and 11 o'clock and two rightward directions are at one and two o'clock. Twelve o'clock is used to represent a straight, forward direction. Object-type messages are defined based on the object classes described in Section 3.4.4. When many objects appear at the same time, the type of every object cannot be announced one by one. Considering that the object closest to the user is the most critical, only the closest object's type is delivered to the user. However, the location of the closest object is difficult to deliver using verbal messages, because it changes from frame to frame. Therefore, an acoustic beeper message set is defined to announce the nearest object position, which is similar to that in [16]. The definition of beeper messages are shown in Figure S3 and Table S2.

### 4.3. Message Delivery Scheme

To define a proper message delivery scheme, the estimated walking context offers valuable cues for selecting the messages that are more important at the moment. The set of message delivery rules is defined in Table 2.

In a “danger” context, it is urgent for the user to receive instantaneous messages about safe walking directions. Less important messages should be blocked in order to avoid their interference with the delivery of messages that give safe directions. In normal, safe contexts, it is more desirable for the user to know the types and positions of objects in the surrounding environment in order to maintain a safe walking direction. Therefore, in safe and normal contexts, a full set of message types can be delivered, whereas in dangerous contexts, only safe-direction and walking context messages are allowed to be delivered.

Another important aspect is the delivery timing. Here, the messages are divided into three types of timing, as shown in Table 3. Hard timing messages have a higher priority than soft timing messages do, and they must be delivered instantly whenever changes in the situation are detected. In hard timing messages, safe direction messages have a higher priority than walking context messages do. User motion messages have the lowest priority in these hard timing message sets.

Messages about the closest object's type are defined as soft timing messages. This type of message is considered less critical for the user's walking safety. Therefore, soft timing messages can be blocked by any hard timing message. Messages about the location of closest object are defined as real timing messages. Because an acoustic beeper is used to indicate object location in real time, this type of beeper message can be delivered in parallel with other verbal messages.

## 5. Experimental Results

To validate the effectiveness of the proposed system, it was tested using real-scene data collected from a laser scanner and a camera. In this section, the experimental results of each part of the system will be shown and discussed.

### 5.1. System Prototype and Test Data Collection

**Figure 20.**
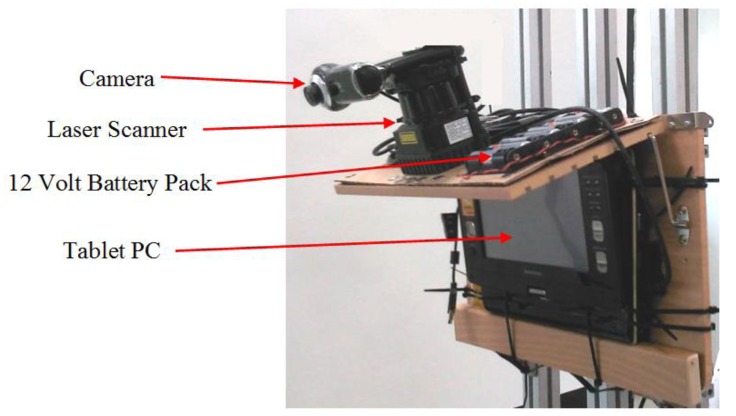
System prototype.

**Figure 21.**
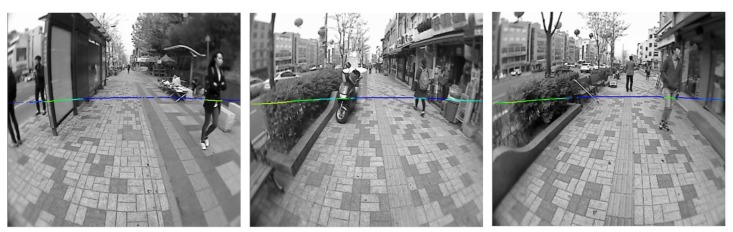

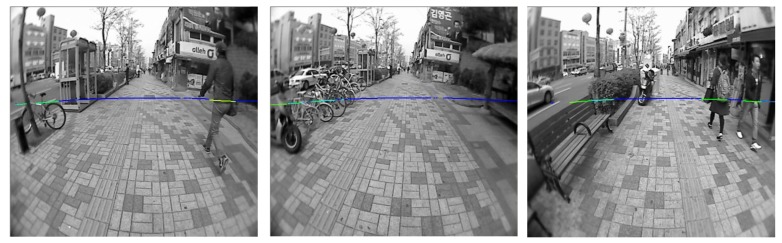
Test data samples.

A prototype of the system is shown in Figure 20. A camera and a laser scanner were bound to each other and fixed on a solid shelf. The combined sensors were connected to a tablet computer for the acquisition and processing of the sensor data. The specifications of the sensors used in the experiment are shown in Table 4. A wearable design of the system prototype is shown Figure S6. In the experiment, the prototype system was mounted on a human who traversed various urban paths. Image frames with synchronized laser scan data on these urban path scenes were collected and recorded. Some test data samples are shown in Figure 21, and the system output on processing these test data is shown in Figure S4 and Figure S5.

### 5.2. Ground Estimation Performance

In this section, the ground estimation performance will be evaluated using the collected test data. Ground estimation is the most essential part of the whole system. It not only gives the ground location, but also provides a reference for generating hypotheses about the objects.

The performance of the ground estimation is analyzed according to two aspects: ground evidence model fitting and ground laser point detection rate. The ground evidence model refers to the Gaussian-uniform mixture type of error model. To evaluate its fitting performance, 13 representative scenarios were selected from test data. These 13 scenarios contained various ground conditions, from flat to rough, and various object densities, from low to high. The mixture type of error model was used to fit the laser scan data of these 13 scenarios. In the first round, the mixture model parameters were fitted from data using the EM algorithm. In the second round, the mixture model parameters were manually tuned to reflect real data distributions. The parameters of the manually tuned mixture model were used as true model parameters in order to evaluate how well model parameters were fitted.

**Figure 22.**
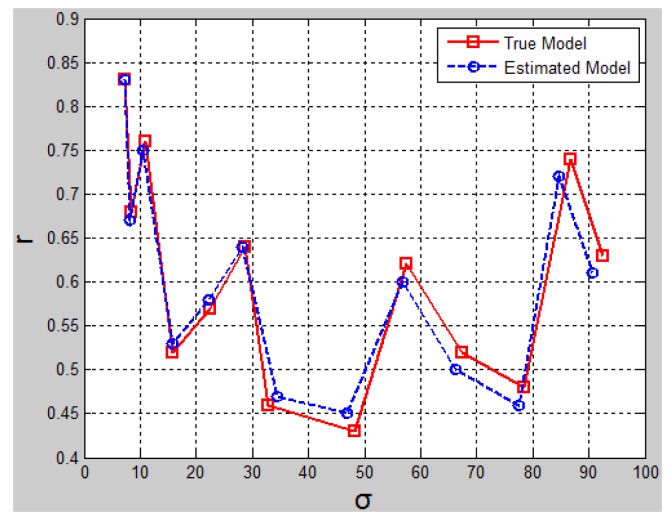
Ground evidence model fitting performance.

In Figure 22, the best fitting parameters (*r*, *ó*) for each scenario in the first round are referred to as the “estimated model” marked with small circles. The parameters of the manually tuned model are referred to as the “true model” marked in small rectangles. Variance *ó* reflects the spreading range of the ground laser points, and the inlier ratio *r* reflects observable ground areas in the scene. It can be observed that in scenarios with small *ó* and large *r*, the estimated model parameters approached true model parameters very closely, whereas in scenarios with large *ó* and small *r*, more obvious deviation can be noticed between the estimated model and the true model.

From the results shown in Figure 22, it can be concluded that the Gaussian-uniform error model reflected the real data distribution well in cases of relatively flat ground with few objects. In cases of rough ground with many objects, the estimated *ó* tended to be smaller than the true *ó* value, which indicates a higher probability of identifying ground laser points as object laser points. This might give rise to the false negative rate in terms of ground laser point detection. Despite some increased inaccuracies in cases of rough ground, the errors were still within the acceptable range.

**Figure 23.**
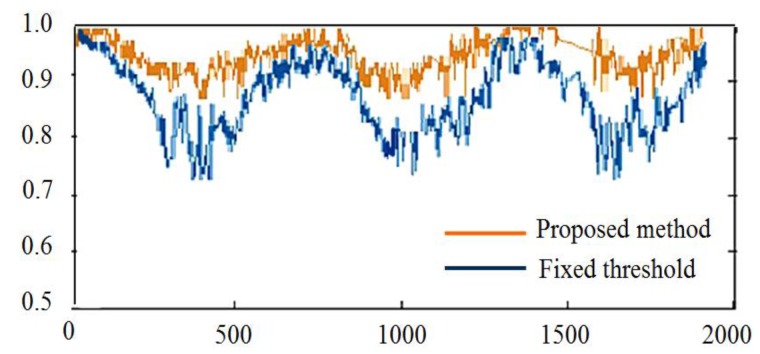
Ground point detection rate.

The ground point detection rate was evaluated using captured video sequence data. The results of detection from a representative video sequence that contains about 1876 data frames are shown in Figure 23. The horizontal axis represents consecutive image frames indexed from zero to 2000, and the vertical axis indicates the true positive rate, which is calculated as in Equation (24), where *TP* is true positives and *FN* is false negatives.

(24) TPR=TP/(TP+FN)

To calculate true positive and false negatives, the laser points of each data frame were manually labeled as ground points or object points. These manually labeled data are used as the ground truth. The results of detection in each data frame were compared with the ground truth; true positives were correctly detected ground points, and false negatives were ground points that were wrongly classified as object points. Of the two types of detection errors (false positive and false negative), here, we are more concerned with false positives. The proposed method tends to produce more false positive errors than false negative errors, which means that there is a higher chance for classifying ground points as object points than *vice versa*. This error producing property is also reasonable for reducing blind user's collision risk.

For a comparison, a method using a fixed threshold to determine ground points and object points was implemented. This fixed threshold method used a fixed distance threshold to the linear ground model to determine whether a laser point was a ground point or not. The true positive rate of ground points detected using the above two methods was calculated for every frame, and the results are shown in Figure 23. The results showed that the proposed method maintained a high detection rate through all frames compared with the fixed threshold method. Although the fixed threshold was tuned to achieve a high detection rate in the first few frames, its detection rate dropped dramatically, then increased and decreased vigorously in a very unstable manner. This result showed that the fixed threshold method could not adapt to accommodate various ground conditions. In contrast, the performance of the proposed method was stable in keeping a high detection rate across all frames.

### 5.3. Object Detection and Classification Performance

**Figure 24.**
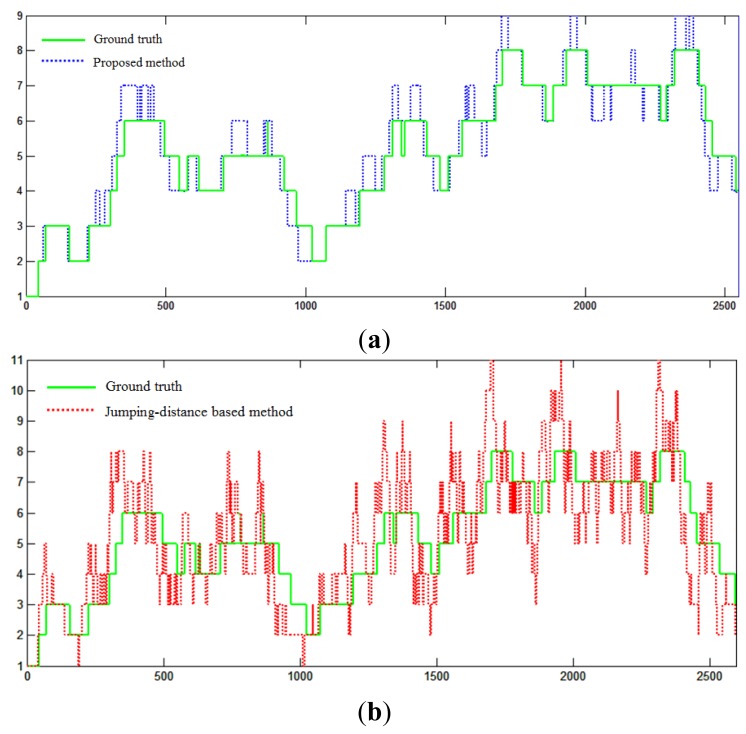
Object detection performance. (**a**) Proposed method performance; (**b**) jumping-distance method performance.

To evaluate the performance of object detection, the number of objects in the test video sequence was used as the major measurement. The results of detection from a representative video sequence containing 2670 data frames are shown in Figure 24a. The horizontal axis shows frame indexes, and the vertical axis shows the number of objects.

The ground truth data were obtained by manually labeling the number of objects in each data frame, and the number of objects detected in each frame was obtained by using the proposed clustering method. As Figure 24a shows, the proposed method tracked the ground truth data well in over 83% of the data frames, which indicates that the proposed smoothness likelihood function was able to capture smooth discontinuities by estimating laser data distributions in a local region.

For a comparison, a jumping distance-based method was implemented. The results of its object detection in the same video sequence are shown in Figure 24b. The jumping distance method used a fixed threshold to segment the laser profiles. As Figure 24b shows, the object detection accuracy of the jumping distance method was much worse than that of proposed method. There are not only large deviations from the ground truth, but also a large variance across frames. The result showed that compared with the proposed method, the jumping distance method tends to produce less accurate and unstable detection results.

**Figure 25.**
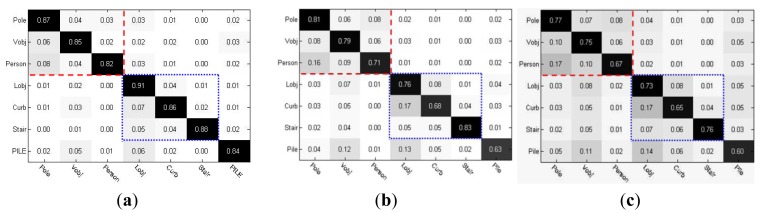
Object classification performance. (**a**) Performance with proposed model; (**b**) Performance without temporal correlation; (**c**) Performance without temporal and spatial correlations.

The object classification performance was evaluated by using the confusion matrix shown in Figure 25. In the confusion matrix, the vertical axis shows the actual category and the horizontal axis shows the predicted category. The object types in each frame of the test video sequence were manually labeled as ground truth data. The results of classification from a representative test video sequence consisting of 2680 frames are shown in Figure 25.

As shown in Figure 25a, the predicted object category labels were produced by using the full multimodal profile model, following the three-layer classification structure. As Figure 25a shows, the classification errors mainly occurred among the object types that belong to the same mid-level category, which is illustrated by the two sub-regions marked in Figure 25a. On the other hand, the confusion rate between the vertical object category and the lateral object category was relatively low. The weighted chain code histogram with temporal accumulation was effective in maintaining a low rate of classification errors in the first layer. This result provided a good basis for identifying finer object classes in the following layers. In fact, the major factor affecting the classification of mid-level categories in the first layer is the partial occlusion and overlapping of object edges in a local region. In the multimodal profile model, we proposed building edge histograms by fusing the edge distributions in multiple local regions both spatially and temporarily to reduce the influence of edge occlusion and overlapping.

To show the effectiveness of this multiple local region scheme, Figure 25b provides the results of classification obtained by using the incomplete multimodal profile model, without the temporal accumulation of the weighted chain-code histogram. Compared with the classification rate shown in Figure 25a, the results showed that the true positive rates in the diagonal dropped, and first-layer classification errors increased, which led to significant error propagation in the second and the third layers of object classes. A chain reaction initiated by the increase in classification errors among the first-layer categories resulted in the increase in total errors, which affected almost all classes.

In addition, Figure 25c shows the classification results using the direct fusion of edge and laser profiles. In this direct fusion method, the edge histogram was built using only a single, local region centered at the break point of the laser profile, without considering the edge distributions of other adjacent local regions or adjacent temporal frames. Built in this way, the edge histogram is much more easily affected when edge profile occlusion or overlapping occurs. The deterioration in the rate of object classification is clearly shown in Figure 25c. The chain reaction of severe error propagation caused a dramatic decrease in the true positive rates, with classification errors spreading to the false positive and false negative elements.

In the proposed three-layer classification structure, more specific object types with complex appearances can be identified using sophisticated image descriptors in the third layer. In the three-layer structure, the object class in the bottom layer benefited from its “parent category” in the upper layer. An example is pedestrian detection: if we use the HOG descriptor to detect pedestrians in the entire image, there will be many false positives, and the detection speed will be slow. However, if we use the multimodal profile model with a three-layer classification structure, the recognition of vertical objects will give reasonable constraints on the possible location of pedestrians. The HOG descriptor can be used to check only several vertical object locations for the recognition of pedestrians. This not only helps reduce false positives, but also hastens the detection of pedestrians.

**Figure 26.**
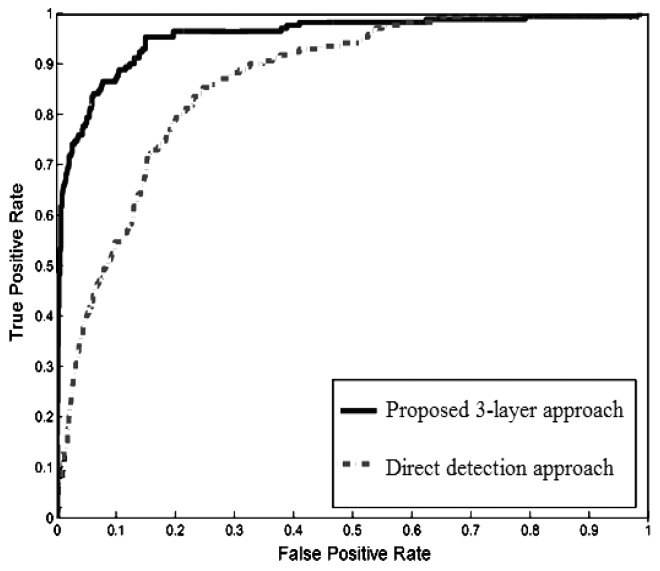
Pedestrian detection performance.

The receiver operating characteristic (ROC) curve of the pedestrian detection rate is shown in Figure 26. The ROC was obtained by using the HOG descriptor with the three-layer structure in the multimodal profile model and by using the HOG descriptor alone to scan the entire image. The results clearly showed that pedestrian detection using the proposed three-layer structure outperforms the direct detection approach.

### 5.4. System Run Time Performance

The entire algorithm was implemented in C++ and tested on a laptop computer. The specifications of the testing platform are listed in Table 5. In Table 6, average runtime is shown with respect to each module, each function in the module, as well as each iteration unit of the function. The iteration unit of each function is shown in Table 7. The runtime of each function is calculated as the average runtime value on test data sets for one frame.

The indicator “Runtime per iteration unit” shows that it takes about 1.2 ms (0.62 ms + 0.58 ms) to recognize one generic object type. Generic object types are those in the first and second layer of the three-layer structure, as shown in Figure 15. For pedestrian detection in the third layer, it takes 4.87 ms to calculate the HOG descriptor and to do the classification for one hypothesized window. When calculating the HOG descriptor, a GPU accelerated version was used, which is much faster than a CPU-based version. The total average run-time was 76.17 ms for one data frame, which is approximately 13 fps.

In our experiment, a blind pedestrian was observed to walk at a speed of around 0.5 m/s∼1.8 m/s on average. Under this walking speed, 3∼5 s was an appropriate time interval for message delivery. Therefore, to meet the run-time requirement of the guidance task, a minimum processing speed of 5 fps was required. With non-optimized code in debugging mode, the proposed system can run at approximately 10∼15 fps. This shows that the proposed system could fully satisfy the real-time requirements for general guidance tasks. For the maximum traveling speed at which the runtime performance of the system is still reasonable, it actually depends on the time it takes to read out one verbal message to the user; because the time it takes to read out one verbal message to the user is much longer than the time it takes for data acquisition and processing. For example, it normally takes 1 s to finish reading one verbal message defined in Table 1. Given that data acquisition and processing costs an additional 0.1 s, then the system response time will add up to 1.1 s. Assuming sensors can detect objects at 5 m ahead of the user, the maximum traveling speed of the user can be estimated as 4.54 m/s. This maximum speed is already over two-times faster than normal pedestrian speed.

## 6. Conclusions

In this paper, an audio guidance system based on context awareness is proposed to assist blind people in traversing local pathways safely and efficiently. The proposed multimodal profile model greatly improved the resolution of scene interpretation by enabling multi-category object identification. An audio message interface was proposed to deliver highly semantic information to the user, based on a walking context. The proposed system has the following limitations. In ground estimation, the proposed ground model worked under the assumption that a sufficient number of ground laser points can be observed and approximated by a linear model. Therefore, in highly crowded situations where the ground area is largely occluded by other objects, ground estimation may not be correct or even fail. The detection of objects, especially low-level objects, relies heavily on ground estimation. If ground estimation is wrong or fails, large errors will also occur in object detection. Future work would include the estimation of sensor motion by fusing visual odometry and IMU data, which would further improve the laser profile tracking and object detection. Localization functions using GPS or image-based scene recognition could also be added to improve the usefulness of the system.

## Supplementary Material



## Figures and Tables

**Table 1.** Message definition.

**Message Type**	**Message Example**
Safe direction	“Go (12 o'clock)”.
Walking context	“Danger context attention”. “Back to normal/safe”.
Closest object type	“vertical object”, “lateral object”, “pole”, “person”, “curb”, “pile”
Closest object location	Acoustic beeper message
User motion	“Please walk faster/slow down/stop”. “Large departure attention”.

**Table 2.** Message delivery rules.

**Walking Context**	**Output Message Set**
Safe	safe direction, user motion close obstacle type and position
Normal	safe direction, user motion, closest obstacle type and position
Danger	safe direction, walking context

**Table 3.** Message delivery timing.

**Types of Timing**	**Instruction Set**
Hard timing	safe direction > walking context > user motion
Soft timing	closest object type
Real timing	closest object position

**Table 4.** Sensor specification.

**System Component**	**Specification**	
Hokuyo Laser Scanner UTM-30LX-EW	Angular resolution	0.25°
	Range resolution	1 mm
	Scanning speed	25 ms/scan
	Scanning range	270°
	Measuring range	0.1 m∼30 m

Logitech Webcam 9000L	Image resolution	640 × 480
	Frame rate	30 fps

**Table 5.** Testing platform specification.

**Platform Component**	**Specifications**
CPU	Intel i5@2.5 GHZ
Memory	4GB DDR3
GPU	GeForce GT 740M
OS	Windows 7 32 bit
Programming Tool	Visual Studio 2010
Compiler	Microsoft VC++ 10

**Table 6.** Average run time performance.

**Module**	**Function**	**Runtime/Iteration Unit**	**Runtime/Function**	**Runtime/Module**
Ground estimation	Ground model fitting	1.03 ms	7.34 ms	7.34 ms

Object detection	Laser profile clustering	0.12 ms	6.52 ms	21.25 ms
	Laser profile tracking	0.83 ms	14.73 ms	

Object classification	Multimodal profile histogram	0.62 ms	8.56 ms	38.83 ms
	Generic object classification	0.58 ms	6.82 ms	
	Pedestrian classification	4.87 ms	23.45 ms	

User feedback	Context estimation	5.35 ms	5.35 ms	8.75 ms
	Message generation	0.86 ms	3.4 ms	

Total	All functions		76.17 ms	76.17 ms

**Table 7.** Iteration unit of each function.

**Function**	**Iteration Unit**
Ground model fitting	One hypothesized model fitting
Laser profile clustering	One point converges to cluster center
Laser profile tracking	Tracking of one laser profile
Multimodal profile histogram	Get multimodal histogram from one object
Generic object classification	Classification of one generic object
Pedestrian classification	Classification of one pedestrian
Context estimation	Estimate context for one frame
Message generation	Generate one message
