# Supplementary material for: A Context-Aware-Based Audio Guidance System for Blind People Using a Multimodal Profile Model
Source: Sensors (Basel). 2014 Oct 9;14(10):18670–700. doi: 10.3390/s141018670 (PMC4239897; doi:10.3390/s141018670)
Supplement: Supplementary file 1 [file sensors-14-18670-s001.pdf]

OPEN ACCESS
sensors
ISSN 1424-8220
www.mdpi.com/journal/sensors

Supplementary Information

A Context-Aware-Based Audio Guidance System for Blind People Using a Multimodal Profile Model. *Sensors* 2014, *14*, 18670-18700

Qing Lin and Youngjoon Han *

Electronic Engineering Department, Soongsil University, 511 Sangdo-Dong, Dongjak-Gu, Seoul 156-743, Korea; E-Mail: lqsdust@163.com

* Author to whom correspondence should be addressed; E-Mail: young@ssu.ac.kr; Tel.: +82-02-820-0699.

1. Fuzzy Rules Used in the Fuzzy Inference Model

Table S1. Fuzzy rules definition.

No.	ρ	d	φ	v	c
1	low	near	small	slow	normal
2	low	near	small	medium	danger
3	low	near	small	fast	danger
4	low	near	medium	slow	normal
5	low	near	medium	medium	danger
6	low	near	medium	Fast	danger
7	low	near	large	any	danger
8	low	medium	small	slow	safe
9	low	medium	small	medium	safe
10	low	medium	small	fast	normal
11	low	medium	medium	slow	normal
12	low	medium	medium	medium	normal
13	low	medium	medium	fast	danger
14	low	medium	large	slow	normal
15	low	medium	large	medium	danger
No.	ρ	d	φ	v	c
16	low	medium	large	fast	danger
17	low	far	small	any	safe
18	low	far	medium	slow	normal
19	low	far	medium	medium	normal
20	low	far	medium	fast	danger

Table S1. *Cont.*

No.	ρ	d	φ	v	c
21	low	far	large	slow	normal
22	low	far	large	medium	normal
23	low	far	large	fast	danger
24	medium	near	small	slow	normal
25	medium	near	small	medium	normal
26	medium	near	small	fast	danger
27	medium	near	medium	any	danger
28	medium	near	larger	any	danger
29	medium	medium	small	any	normal
30	medium	medium	medium	slow	normal
31	medium	medium	medium	medium	normal
32	medium	medium	medium	fast	danger
33	medium	medium	larger	slow	normal
34	medium	medium	larger	medium	danger
35	medium	medium	larger	fast	danger
36	medium	far	small	slow	safe
37	medium	far	small	medium	safe
38	medium	far	small	fast	normal
39	medium	far	medium	any	normal
40	medium	far	large	slow	normal
41	medium	far	large	medium	normal
42	medium	far	large	fast	danger
43	high	near	small	slow	normal
44	high	near	small	medium	danger
45	high	near	small	fast	danger
46	high	near	medium	any	danger
No.	ρ	d	φ	v	c
47	high	near	large	any	danger
48	high	medium	small	any	normal
49	high	medium	medium	slow	normal
50	high	medium	medium	medium	danger
51	high	medium	medium	fast	danger
52	high	medium	large	any	danger
53	high	far	small	slow	safe
54	high	far	small	medium	normal
55	high	far	small	fast	normal
56	high	far	medium	slow	safe
57	high	far	medium	medium	normal
58	high	far	medium	fast	normal
59	high	far	large	slow	normal
60	high	far	large	medium	normal
61	high	far	large	fast	danger

2. A Fuzzy Inference Process for Walking Context

Figure S1. An example of fuzzy inference process.

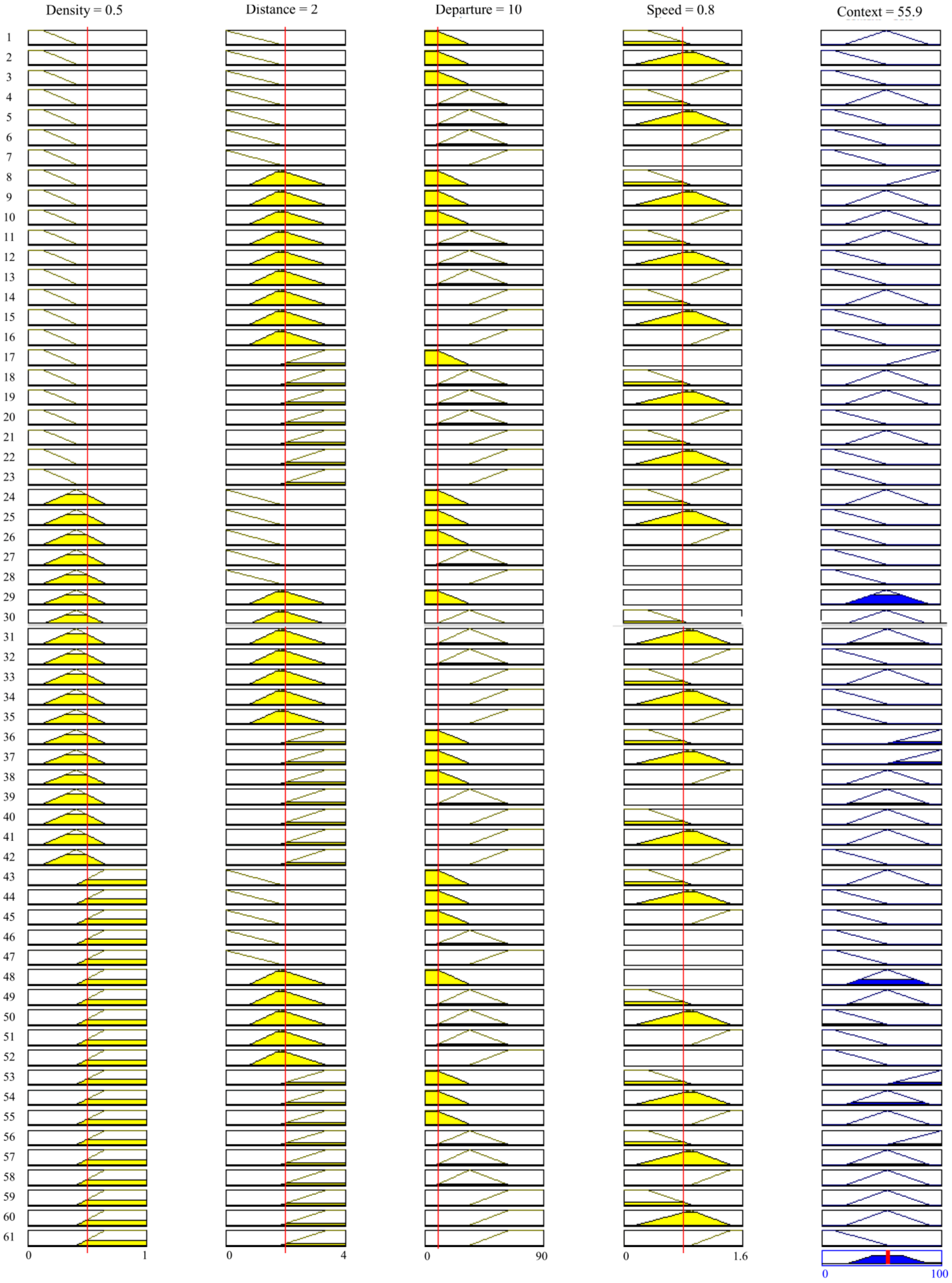

3. Walking Context Fuzzy Inference Solution Space

Figure S2. Fuzzy inference solution space.

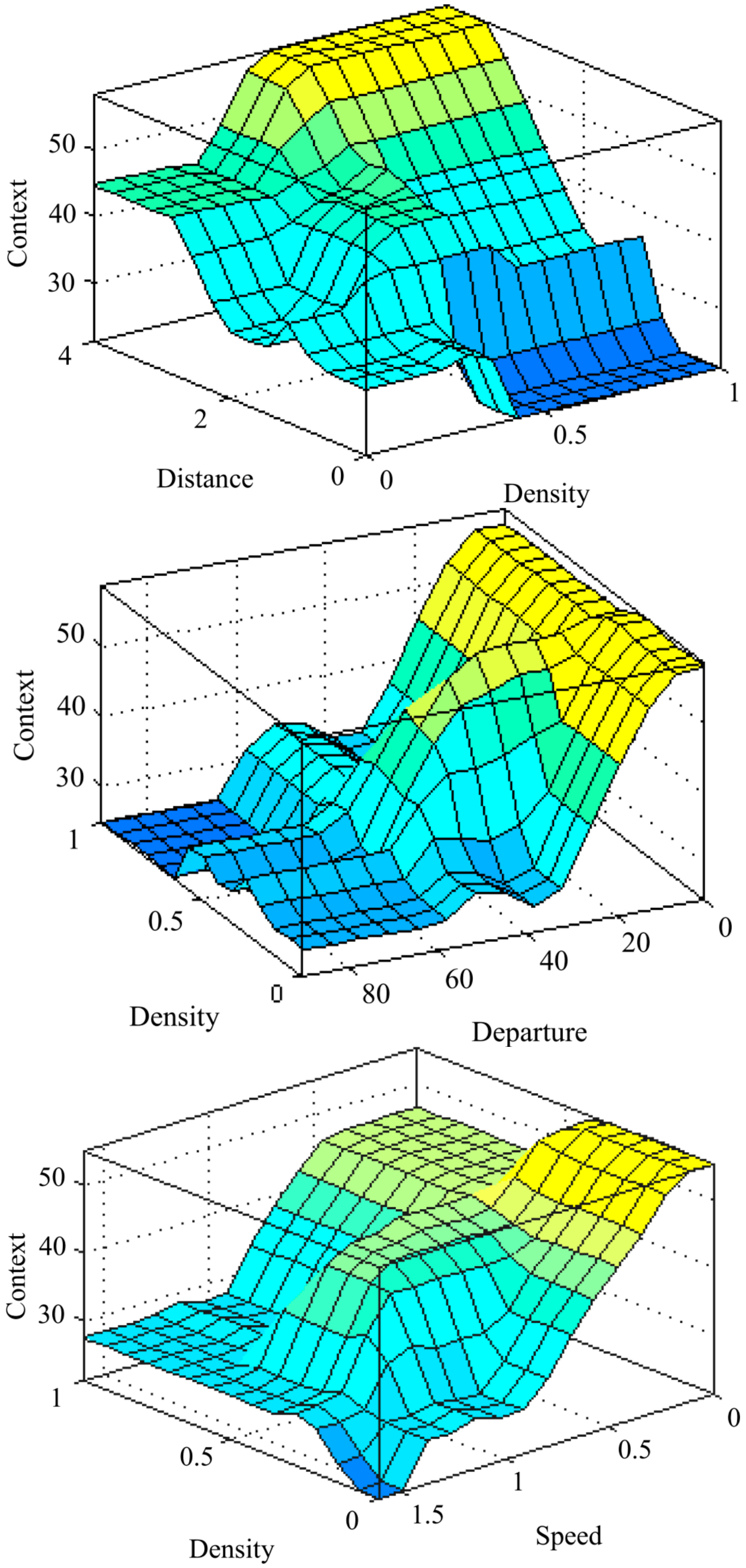

Figure S2. *Cont.*

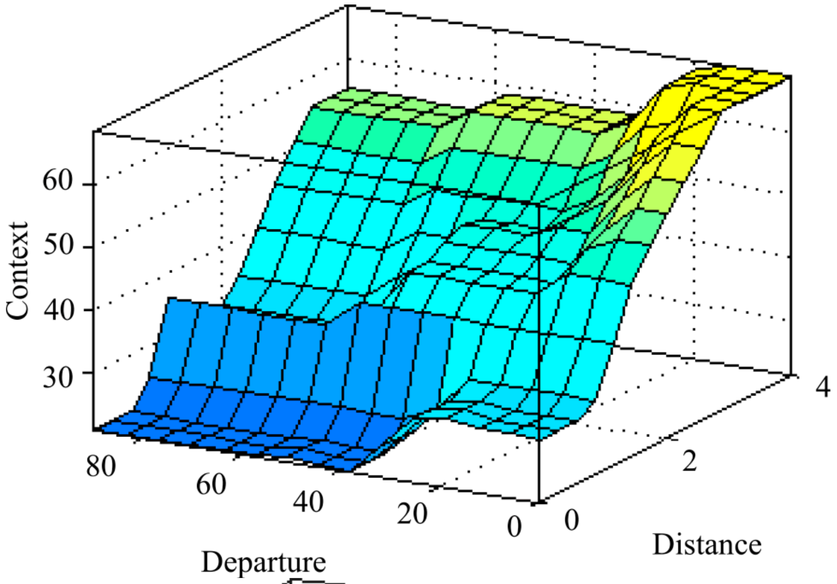

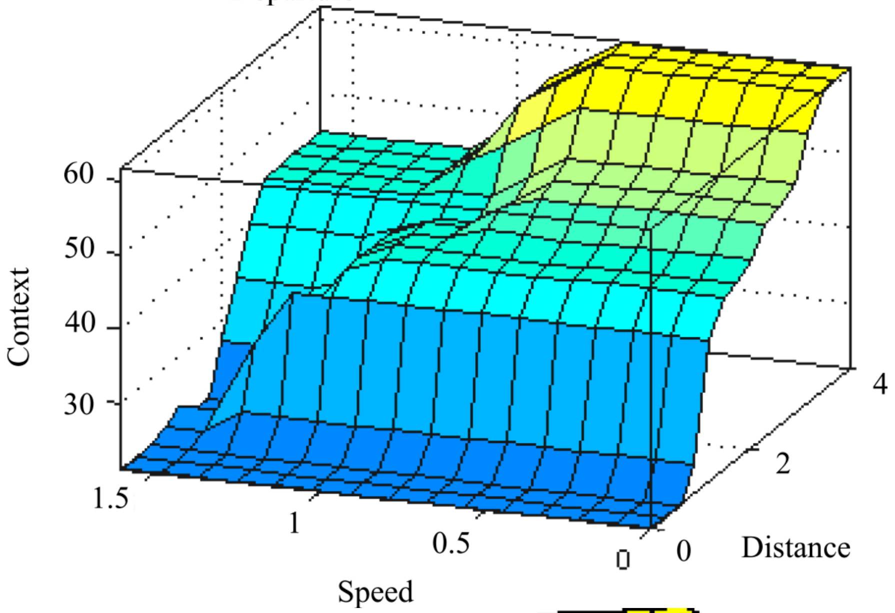

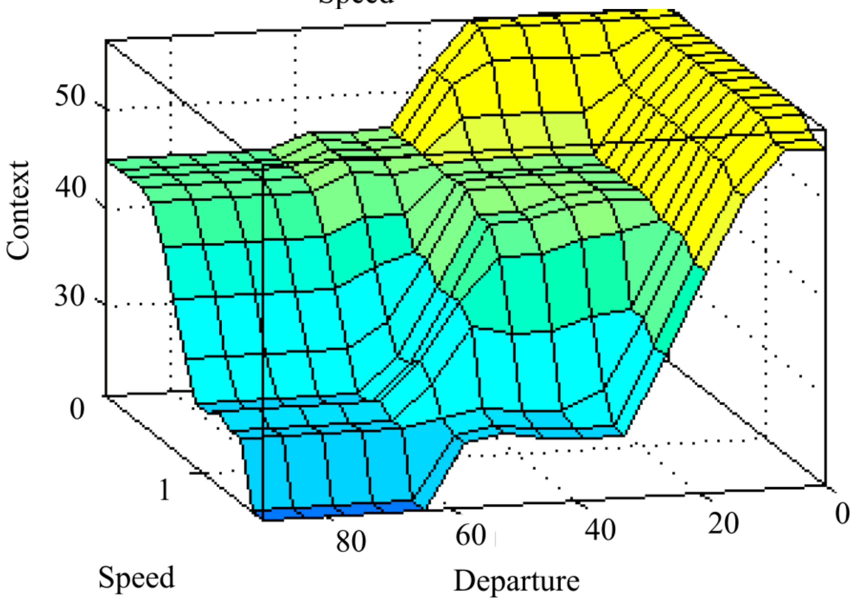

4. Acoustic Beeper Message Definition

Figure S3. Beeper message definition using polar array.

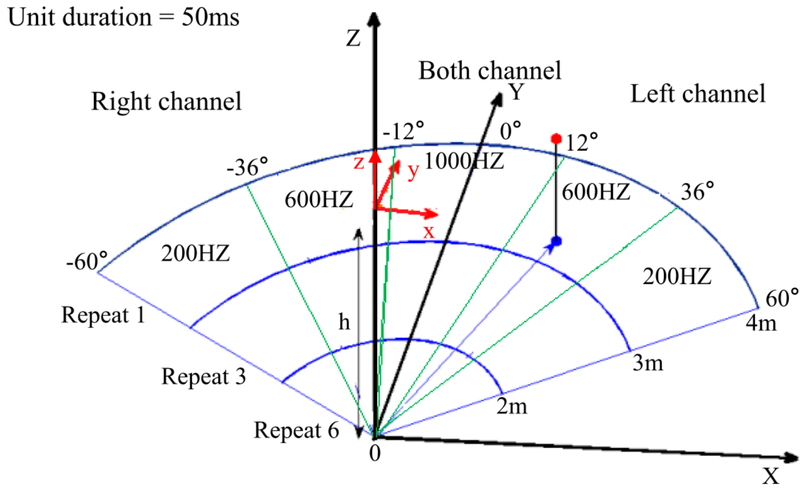

Table S2. Beeper message definition in different polar zones.

	**<2 m**	**2 m ~ 3 m**	**3 m ~ 4 m**
−12° ~ 12°<br>Center Zone	F = 1000 HZ, L = 50 ms,<br>Repeat = 6, Channel = both	F = 1000 HZ, L = 50 ms,<br>Repeat = 3, Channel = both	F = 1000 HZ, L = 50 ms,<br>Repeat = 1, Channel = both
12° ~ 36°<br>Right-1 Zone	F = 600 HZ, L = 50 ms,<br>Repeat = 6, Channel = right	F = 600 HZ, L = 50 ms,<br>Repeat = 3, Channel = right	F = 600 HZ, L = 50 ms,<br>Repeat = 1, Channel = right
36° ~ 60°<br>Right-2 Zone	F = 200 HZ, L = 50 ms,<br>Repeat = 6, Channel = right	F = 200 HZ, L = 50 ms,<br>Repeat = 3, Channel = right	F = 200 HZ, L = 50 ms,<br>Repeat = 1, Channel = right
−12° ~ −36°<br>Left-1 Zone	F = 600 HZ, L = 50 ms,<br>Repeat = 6, Channel = left	F = 600 HZ, L = 50 ms,<br>Repeat = 3, Channel = left	F = 600 HZ, L = 50 ms,<br>Repeat = 1, Channel = left
−36° ~ −60°<br>Left-2 Zone	F = 200 HZ, L = 50 ms,<br>Repeat = 6, Channel = left	F = 200 HZ, L = 50 ms,<br>Repeat = 3, Channel = left	F = 200 HZ, L = 50 ms,<br>Repeat = 1, Channel = left

5. Guidance System Output

Figure S4. System output in image domain.

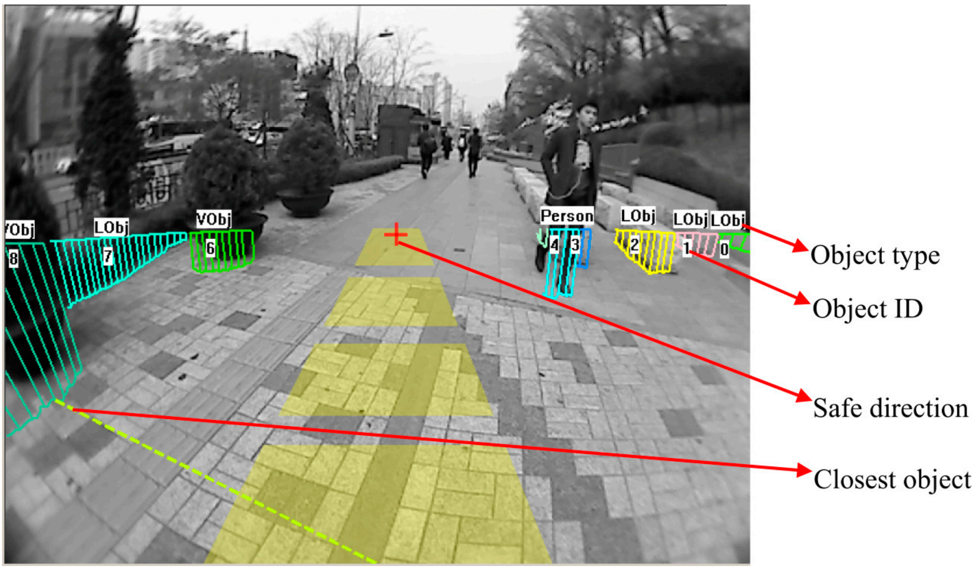

Figure S5. System output in laser domain.

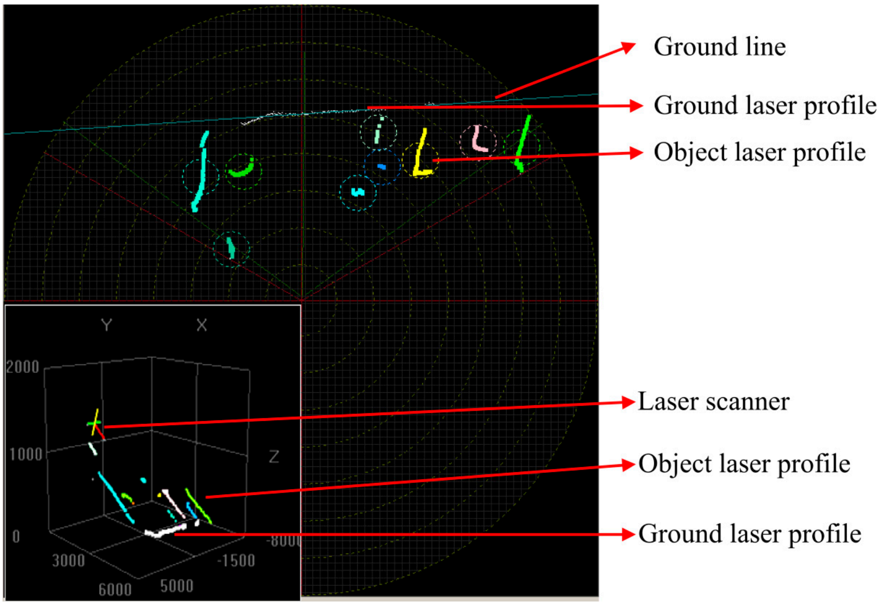

Please refer to: http://youtu.be/c5AcJhNXgj0 for a demo video of the system.

6. System Prototype

Figure S6. Prototype system.

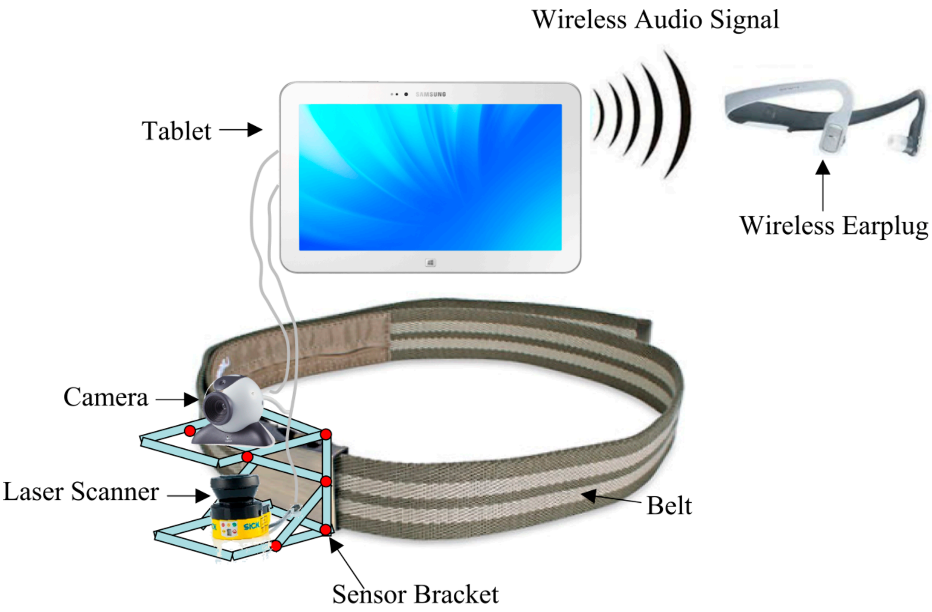

Table S3. Prototype system specification.

Prototype Part	Size (L × W × H) mm	Weight
HOKUYO URG laser scanner	50 × 50 × 70	160 g
Microsoft LifeCam VX-800	52 × 52 × 62	68 g
Sensor bracket	60 × 60 × 80	105 g
Samsung ATIV Tab 3	257.4 × 165.3 × 8.2	550 g
Iwoo Sports Headset	Adjustable to head	60 g
Total		943 g

As shown in Figure S6, the prototype system is built as a wearable belt. Camera and laser scanner are mounted on a sensor bracket attached to the belt. The camera and laser scanner are connected to a tablet PC via USB cables. The generated audio messages are forwarded to the user via a wireless earplug. The weight and size of each part of the prototype system are listed in Table S3. The total weight is less than 1 kg.

For power consumption, both the webcam and laser scanner draw power via USB cable from the tablet PC. Samsung ATIV Tab 3 comes with a built-in 2 cell battery capable of offering 25.6 Wh power. The specification of Samsung ATIV Tab 3 is listed in Table S4, and the power consumption of major parts is listed in Table S5. When running the system algorithm on a full working load, the whole system consumes about 8.6 W, and when system is in standby mode, it only consumes 2.2 W.

Table S4. Samsung ATIV Tab 3 Specification.

Tablet Part	Specification
CPU/Chipset	Intel® ATOM™ Processor Z2760 (1.8 GHz, 1MB L2 Cache)
Memory	2 GB LPDDR2 System Memory at 800 MHz
Graphic	Intel® Graphics Media Accelerator
Storage	64 GB e.MMC iNAND™ Embedded Flash Drive
Display	10.1" SuperBright + 400nit HD LED Display (1366 × 768)
Power	2 Cell Li-ion Battery (25.6 Wh)

Table S5. Power consumption of the prototype system.

Power Consumption Part	Power Consumption
Tablet platform	4.5 W(working)/2.2 W(standby)
Laser scanner	2.5 W
Webcam	1.6 W
Total	8.6 W(working)/2.2 W(standby)

Figure S7. System working hours.

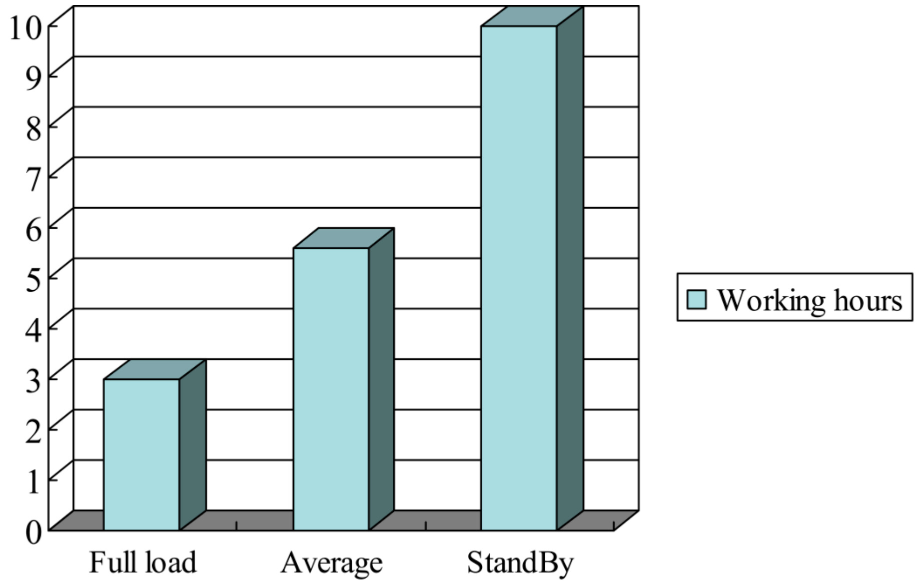

In Figure S7, the working hour of system powered by a 25.6 Wh Li-ion battery is listed. When working on full load, the system can hold for around 3 h. And in standby mode, the system can last for almost 10 h. For an average daily use, the system can sustain about 5~6 h on a full charge.

In addition, we are also making embedded board specially designed for running the application. The specially designed board will have smaller size, less weight and lower power consumption compared with a generally purpose tablet that is used in the prototype system.

© 2014 by the authors; licensee MDPI, Basel, Switzerland. This article is an open access article distributed under the terms and conditions of the Creative Commons Attribution license (http://creativecommons.org/licenses/by/4.0/).
